# Exploring the phylogeography of a hexaploid freshwater fish by RAD sequencing

**DOI:** 10.1002/ece3.3821

**Published:** 2018-01-28

**Authors:** Cora Sabriel Stobie, Carel J. Oosthuizen, Michael J. Cunningham, Paulette Bloomer

**Affiliations:** ^1^ Molecular Ecology and Evolution Programme Department of Genetics University of Pretoria Pretoria South Africa

**Keywords:** genotyping‐by‐sequencing, polyploidy, population genomics, population history

## Abstract

The KwaZulu‐Natal yellowfish (*Labeobarbus natalensis*) is an abundant cyprinid, endemic to KwaZulu‐Natal Province, South Africa. In this study, we developed a single‐nucleotide polymorphism (SNP) dataset from double‐digest restriction site‐associated DNA (ddRAD) sequencing of samples across the distribution. We addressed several hidden challenges, primarily focusing on proper filtering of RAD data and selecting optimal parameters for data processing in polyploid lineages. We used the resulting high‐quality SNP dataset to investigate the population genetic structure of *L. natalensis*. A small number of mitochondrial markers present in these data had disproportionate influence on the recovered genetic structure. The presence of singleton SNPs also confounded genetic structure. We found a well‐supported division into northern and southern lineages, with further subdivision into five populations, one of which reflects north–south admixture. Approximate Bayesian Computation scenario testing supported a scenario where an ancestral population diverged into northern and southern lineages, which then diverged to yield the current five populations. All river systems showed similar levels of genetic diversity, which appears unrelated to drainage system size. Nucleotide diversity was highest in the smallest river system, the Mbokodweni, which, together with adjacent small coastal systems, should be considered as a key catchment for conservation.

## Graphical abstract

We present the development of a high‐quality SNP panel from double‐digest restriction site‐associated DNA (ddRAD) sequencing of a hexaploid freshwater fish, *Labeobarbus natalensis*. We use the SNP dataset to investigate the phylogeography of this species across the distribution and delimit it into five major populations grouped by drainage system. We encountered a number of unexpected challenges when working with this dataset, emphasizing the need for proper filtering of RAD datasets and optimization of parameter selection.

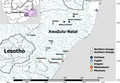

## 1 INTRODUCTION

The Cyprinidae is the largest freshwater fish family, comprising approximately 80% of all freshwater fish species in temperate zones (Naran, Skelton, & Villet, 2007) and including over 2,400 species (de Graaf, Nagelkerke, Palstra, & Sibbing, 2010; Swartz, Mwale, & Hanner, 2008). Within Cyprinidae, the African genus *Labeobarbus* remains relatively understudied. This genus has recently been grouped into the tribe Torini (Yang et al., 2015). *Labeobarbus* is thought to have arisen from hybridization between a tetraploid ancestor in Torini and a diploid ancestor of *Cyprinion* followed by autopolyploidization, resulting in the current hexaploid lineage (2N = ±150 chromosomes) sometime prior to their colonization of Africa *c*. 13 mya (Oellermann & Skelton, 1990; Tsigenopoulos, Kasapidis, & Berrebi, 2010; Yang et al., 2015). This lineage has subsequently speciated into 125 valid species (Vreven, Musschoot, Snoeks, & Schliewen, 2016).

Seven species of *Labeobarbus* exist in southern Africa. Five of these (*Labeobarbus aeneus*,* Labeobarbus capensis*,* Labeobarbus kimberleyensis*,* L. natalensis*, and *Labeobarbus polylepis*) likely originated from a common ancestor invading the Orange River Basin *c*. 2–3 mya (Skelton, 1986). Major geological events *c*. 5.1 mya resulted in deep riverine valleys separating current drainage systems across KwaZulu‐Natal Province of South Africa (Partridge & Maud, 2000; Rivers‐Moore, Goodman, & Nkosi, 2007) which were later colonized by ancestors of the endemic KwaZulu‐Natal yellowfish, *L. natalensis* de Castelnau, 1861. The prevalence of physical barriers such as waterfalls doubtless affected this process, restricting freshwater fish movement or leading to unidirectional movement. This, combined with the stenohaline nature of the fish, make it difficult to understand the dispersal pathways resulting in the now widespread occurrence of the species in the KwaZulu‐Natal rivers.

Despite the current IUCN Red List assessment of *L. natalensis* as least concern (Cambray, Bills, Chakona, Coetzer, & Weyl, 2017), the species may be declining (Karssing, 2008). The genus is highly popular in South Africa both for subsistence and recreational anglers (Skelton & Bills, 2008) and is also used as an indicator of river health—their presence showing low water pollution and few alien fish species (Skelton & Bills, 2008). As such, conservation management is needed for this “flagship” species for freshwater systems (Skelton & Bills, 2008). This should include quantifying the genetic diversity of populations across the species’ geographic range (Palumbi, 2003; Smith & Bermingham, 2005). Various phylogeographic studies have been conducted on cyprinids (Durand, Tsigenopoulos, Ünlü, & Berrebi, 2002; Machordom & Doadrio, 2001) although few of these have explored the South African branches of the family (but see Chakona, Swartz, & Gouws, 2013; Chakona, Malherbe, Gouws, & Swartz, 2015; Chakona & Skelton, 2017; Swartz, Skelton, & Bloomer, 2007, 2009; Swartz, Chakona, Skelton, & Bloomer, 2014; van der Walt, Swartz, Woodford, & Weyl, 2017).

Previous analyses based on mitochondrial DNA (mtDNA) data showed substantial differences between populations of *L. natalensis* across its distribution (Bloomer et al., 2007; Bloomer et al. Unpublished data). More variation was reported between these populations than between two other South African species, *L. aeneus* and *L. kimberleyensis* (Bloomer et al., 2007). Six primary mitochondrial haplogroups were identified for *L. natalensis*, matching major drainage systems—from north to south: the Umfolozi, Tugela, Umgeni, Mbokodweni, Mkomaas, and Mzimkhulu systems (Bloomer et al. Unpublished data). This suggests historical isolation among these drainage systems. The most notable divide was between the northern and southern drainage systems. This disjunction does not correspond closely with any known biogeographic transition. In general, KwaZulu‐Natal has a rich and geographically varied freshwater fauna, but this diversity occurs as a complex regional mosaic, reflecting historical interchange among tropical and temperate faunal elements with substantial local endemism (Perera, Ratnayake‐Perera, & Proches, 2011; Rivers‐Moore et al., 2007). The initial *L*. *natalensis* phylogeographic study was based entirely on mitochondrial markers and thus remains to be verified with genomic data. The processes that may have resulted in genetic structure also remain to be identified.

At present, there is no close reference genome for *Labeobarbus*, in which ancestral hexaploidy has resulted in large and highly paralogous genomes. Consequently, we decided to use a reduced representation approach, restriction site‐associated DNA (RAD) sequencing (Baird et al., 2008; Miller, Dunham, Amores, Cresko, & Johnson, 2007), to understanding genomic diversity in *L. natalensis*. This method is popular and has been used in many studies since its inception (Figure S1). RAD sequencing has been used, particularly in fish, to identify population divergence (Boehm, Waldman, Robinson, & Hickerson, 2015; Ferchaud & Hansen, 2016; Larson et al., 2014), for SNP identification in polyploid fish (Hohenlohe, Amish, Catchen, Allendorf, & Luikart, 2011; Ogden et al., 2013; Palti et al., 2014), in phylogeographic studies (Macher et al., 2015; Reitzel, Herrera, Layden, Martindale, & Shank, 2013), for QTL analysis (Gagnaire, Normandeau, Pavey, & Bernatchez, 2013; Houston et al., 2012; Yoshizawa et al., 2015), for linkage mapping (Brieuc, Waters, Seeb, & Naish, 2014; Henning, Lee, Franchini, & Meyer, 2014), in hybridization studies (Hand et al., 2015; Lamer et al., 2014; Pujolar et al., 2014), for exploration of genome architecture and evolution (Brawand et al., 2014; Kai et al., 2014; Waples, Seeb, & Seeb, 2016), and in phylogenetic analyses (Gonen, Bishop, & Houston, 2015; Wagner et al., 2013). This methodology should be particularly suited to phylogeographic studies as the inference power from large numbers of markers may identify patterns that are not easily visible in traditional analyses based on relatively few loci (Davey et al., 2011). Quality control is critical for RAD sequencing analyses and is conducted at various stages via an analytical pipeline prior to interpreting results for meaningful biological relationships (Davey et al., 2013).

Double‐digest RAD (ddRAD) sequencing (Peterson, Weber, Kay, Fisher, & Hoekstra, 2012) addresses several coverage issues in the original RAD protocol by replacing random shearing of fragments with a second restriction enzyme. Targeted fragments are defined on one end by a common restriction site as in standard RAD sequencing, but differ in being flanked by a less common restriction site at the other end (Peterson et al., 2012). This approach results in higher repeatability, better control over genome coverage, greater sharing of sequenced fragments, and similar sequence read proportions across individuals (Peterson et al., 2012). The additional restriction digestion may also introduce artifacts; however, as mutations in restriction sites may result in underestimation of diversity due to allele dropout (Arnold, Corbett‐Detig, Hartl, & Bomblies, 2013). Indels, combined with stringent size selection, may also result in loci being dropped or included in particular individuals or populations during ddRAD sequencing (DaCosta & Sorenson, 2014).

Polyploidy complicates most genetic analyses of *Labeobarbus*. Many studies of polyploids advocate analysis of non‐nuclear markers or the transcriptome (Everett, Grau, & Seeb, 2011). However, a number of studies using RAD sequencing have recently tackled the challenge, particularly in tetraploid fish. Several strategies have emerged to circumvent the complicating issue of paralogy (reviewed in McKinney, Waples, Seeb, & Seeb, 2017). These include removing diallelic markers yielding more than two alleles or haplotypes per individual and excluding loci where more than half the individuals genotyped appear heterozygous (Hohenlohe et al., 2011, 2013). Recently, McKinney et al. (2017) suggested the Hdplot approach, which compares heterozygosity at each diallelic locus across a population with read depth for each allele.

In this study, we used ddRAD sequencing of samples from across the distribution of *L. natalensis* to identify phylogeographic patterns and processes affecting this species. To do this, we developed a pipeline to filter sequencing artifacts and paralogs from diallelic SNP data, resulting in a high‐quality genomic resource for use in *Labeobarbus*.

## 2 MATERIALS AND METHODS

### 2.1 Sample collection and DNA extraction

**Figure 1:**
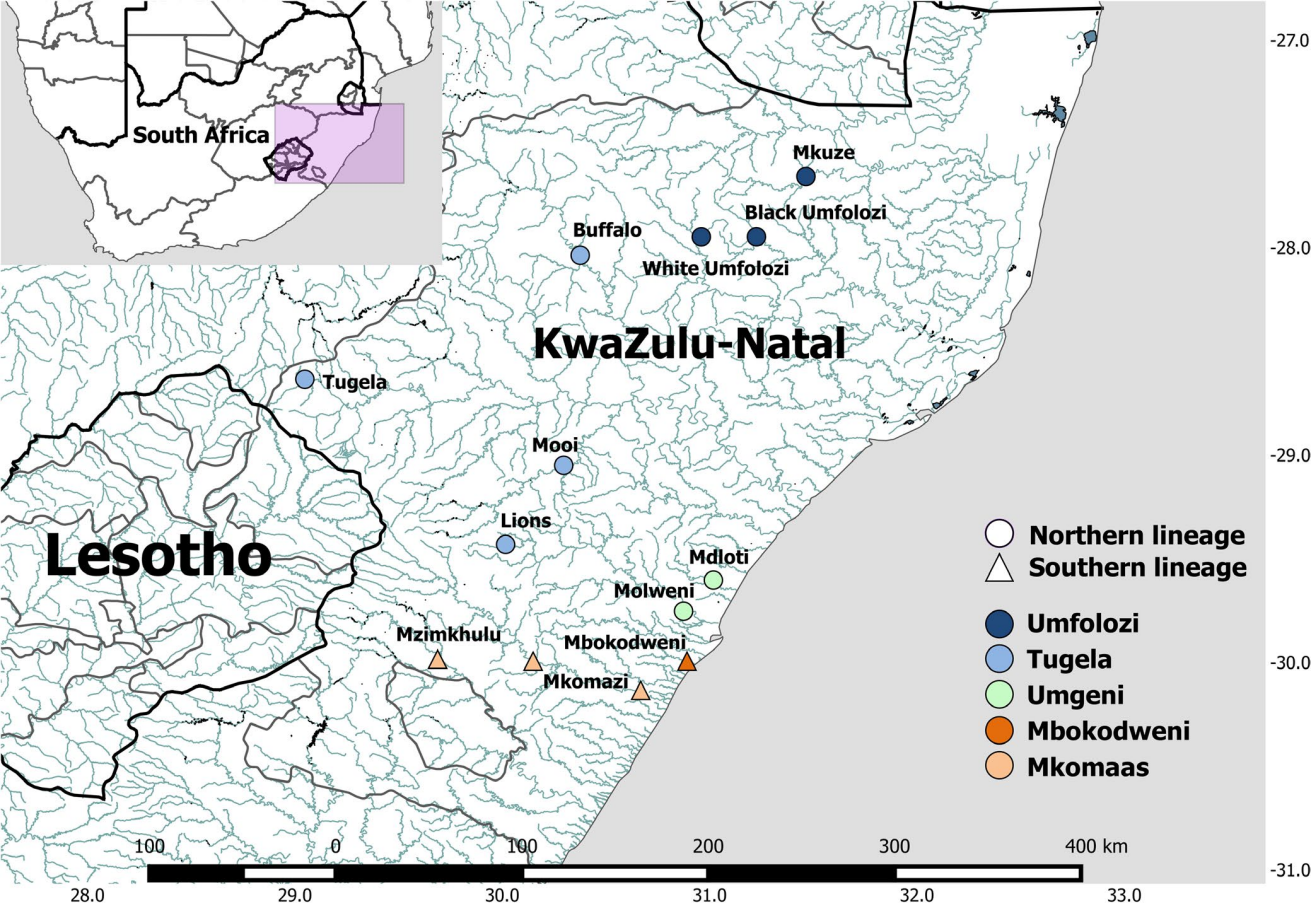
Distribution of samples in this study across KwaZulu‐Natal with reference to a map of South Africa (top left). River names are indicated at each sampling site. The color of each sampling site corresponds to the putative population identified in this study. The shape of the symbol indicates an association to either the northern or the southern lineage. This map was produced using QGIS (QGIS Development Team, 2016. QGIS Geographic Information System. Open Source Geospatial Foundation Project. http://www.qgis.org/) and the National Freshwater Ecosystem Priority Areas (NFEPA) project (Nel et al., 2011)

Samples were collected from 13 localities across the KwaZulu‐Natal Province, South Africa (Figure 1; Table S1) between March 2003 and November 2007. The localities selected represented most major drainage systems across the species distribution. Muscle and fin samples were obtained and stored in 96% ethanol at 4°C. DNA was extracted using the phenol–chloroform method (Sambrook, Fritsch, & Maniatis, 1989).

A GeneQuant™ *pro* RNA/DNA calculator spectrophotometer (Amersham Biosciences, Freiberg, Germany), Qubit^®^ 2.0 Fluorometer (Invitrogen, USA), and agarose gel electrophoresis were used to assess DNA quality and concentration. High‐quality samples were sent to Beijing Genomics Institute Hong Kong Co., Limited (BGI, Hong Kong) to undergo the ddRAD sequencing protocol as per Peterson et al. (2012). In total, 23 high‐quality samples were selected for analysis (Table S1).

### 2.2 Library preparation and sequencing

Samples were divided into two libraries, which were digested with the restriction enzymes *Nla*III (CATG/) and *Mlu*CI (/AATT) following the double‐digest paired‐end protocol of Peterson et al. (2012). Each individual was tagged with a unique 4–8 base pair barcode, with two replicate individuals barcoded and sequenced in separate libraries, as controls. Each library was size selected for fragments of 200–400 bp. Final libraries were sequenced using 90‐bp paired‐end sequencing in a single lane of an Illumina HiSeq 2000 (Illumina Inc., USA). The resulting reads were screened for poor quality (reads with more than 50% low‐quality bases i.e., quality value ≤ 5 (E)), trimmed to remove adapters and barcodes, and demultiplexed prior to analysis.

### 2.3 Bioinformatics pipeline, quality control and read mapping

Data were cleaned both with custom bioinformatic expressions and using the program process_radtags in stacks 1.44 (Catchen, Amores, Hohenlohe, Cresko, & Postlethwait, 2011; Catchen, Hohenlohe, Bassham, Amores, & Cresko, 2013). Reads were first trimmed to a uniform read length of 80 bp to reduce the effect of sequencing error, after examination of SNP density spectra generated from the untrimmed data (Figure S2). Quality of the bases was assessed for each sample both before and after trimming using the program fastQC (Andrews, 2010; Figures S3 and S4). After trimming, the parameters *−r* (rescue RAD tags), *−c* (clean data, remove reads with an uncalled base), and *−q* (remove low‐quality reads) were specified in process_radtags. Only reads that remained paired after processing were retained. Adapter pollution, remnants of adapter sequences that were not removed by earlier trimming, was filtered using custom bioinformatic expressions. The degree of overlapping reads and incomplete restriction digestion in the dataset was estimated using custom bioinformatic expressions and CLC genomics workbench 7.0.4 (CLC Inc., Aarhus, Denmark). The latter program was also used to evaluate read quality and to map filtered reads to the closest cyprinid reference genome, the common carp (*Cyprinus carpio*, GCF_000951615.1). Filtered reads were similarly mapped to the set of carp coding DNA sequences (CDS) from the same reference after removing mitochondrial genes. We used a mismatch cost of 2, insertion and deletion costs of 3, and length fractions and similarity fractions of 0.9 to retain only highly plausible mappings. Nonspecific matches were ignored.

### 2.4 Single‐nucleotide polymorphism discovery

**Figure 2:**
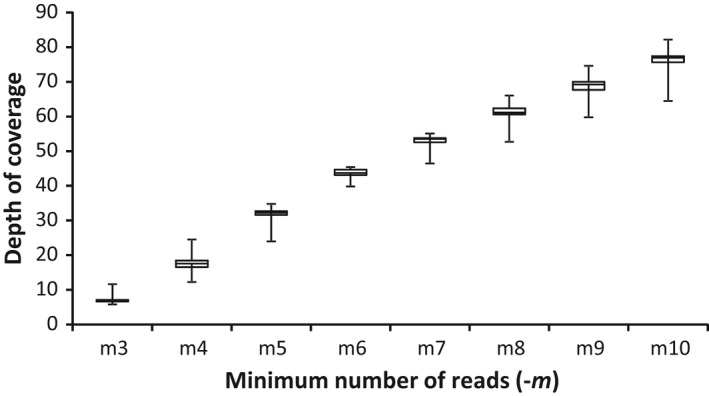
Box‐and‐whisker plot showing the distribution of the mean depth of coverage across all 23 samples (plus two replicates) as the value for the minimum number of reads (−*m*) is varied from 3 to 10, as per Paris et al. (2017). Whiskers here indicate the maximum and minimum values across the samples. Paris et al. (2017) recommend a depth of coverage of >25×, which would indicate that −*m *=* *5 is most suitable for this dataset. The default parameter set was −*m* 5 −*M* 3 −*n* 2

Due to the lack of a close reference genome, the wrapper program denovo_map.pl was used to identify diallelic SNP loci by executing ustacks, cstacks, and sstacks (Catchen et al., 2011, 2013). We chose to use only our Read 1 files (*Nla*III cut‐site) for the assembly to avoid duplication of SNPs due to overlapping reads from fragment ends and false SNPs caused by adapter pollution. Optimal parameters were identified using the method outlined in Paris, Stevens, and Catchen (2017). Briefly, we constructed plots of the average read depth across samples while varying the minimum stack depth parameter *−m* (Figure 2). We then compared the number of SNPs, assembled loci, and polymorphic loci for each sample and across samples using the 80% sample representation cutoff suggested by Paris et al. (2017) while varying the minimum stack depth (*−m*) and distance allowed between stacks (*−M*) from defaults of *−m* 5 *−M* 3 (Figures S5 and S6). Finally, the maximum distance required to merge catalog loci (*−n*) was assessed by evaluating the change in number of polymorphic loci for *n* = *M*−1, *n* = *M*, and *n* = *M *+* *1 (Table S2). The *‐‐max_locus_stacks* default value was set to 7 to ensure adequate binning and avoid paralogs. The −*t* flag was specified while running denovo_map.pl to remove or split highly repetitive tags during ustacks. From this procedure, we identified optimal parameters for this dataset to be −*m* 5 −*M* 1 −*n* 0.

Mitochondrial reads were detected using nucleotide BLAST (Altschul et al., 1997) to match (*E *≤* *1 × 10^−20^) against the available mitogenomes for five related genera*—Labeobarbus* (*L. intermedius* NC_031531.1; *L. *sp. Kongou AP011324.1; *L. *sp. Lucien AP011323.1), *Varicorhinus* (NC_031528.1), *Tor* (NC_027617.1; KU870466.1; NC_021755.1; AP011326.1; NC_022702.1; JX444718.1; AP011372.1; KR868704.1), *Neolissochilus* (NC_026106.1; KU553349.1; AP011314.1; NC_031555.1), and *Hypselobarbus* (NC_031587.1). We removed potential paralogs that had been merged by identifying loci that possessed more than two haplotypes within a single sample. We compared this haplotype approach with the Hdplot method of McKinney et al. (2017). A blacklist was constructed in populations for loci identified as mitochondrial or paralogous.

We retained loci from populations that were scored in at least 60% of all individuals. We used a minor allele frequency (MAF) filter of 0.04 to filter out singleton SNPs that may mask population structure (Rodríguez‐Ezpeleta et al., 2016; Roesti, Salzburger, & Berner, 2012), and a maximum observed heterozygosity filter of 0.99 to remove SNPs that were reported as heterozygotes in all samples the SNP was called in, which are potentially paralogous loci. Finally, because some analyses required a single SNP per locus, we filtered our dataset by selecting the most informative SNP per locus based on the number of minor alleles. Where multiple SNPs at a locus had the same number of minor alleles, we chose the first SNP with the best representation across samples. Loci that passed all filtering criteria were extracted using a whitelist and run through populations again to produce the final dataset of 723 SNPs, which was used in all downstream analyses unless specified otherwise.

### 2.5 Population genetic parameters and structure

Output from populations was exported in STRUCTURE and GENEPOP formats and converted to other formats, as needed, using PGD spider (Lischer & Excoffier, 2012). STRUCTURE 2.3.4 (Pritchard, Stephens, & Donnelly, 2000) was used to infer population structure with 100,000 chains as burn‐in and 500,000 MCMC chains with 20 iterations for *K *=* *1–8. Location was specified as a prior. We followed the same protocol for further hierarchical STRUCTURE runs for the two lineages identified from the primary run. The result files were run through STRUCTURE harvester (Earl & VonHoldt, 2011), and the optimal *K*‐value was determined by the method of Evanno, Regnaut, and Goudet (2005). CLUMPP 1.1.2 (Jakobsson & Rosenberg, 2007) was used to visualize the data.

In addition to STRUCTURE, RAdpainter and fineRAdstructure (Malinsky, Trucchi, Lawson, & Falush, 2016) were used as an independent assessment of population structure, as this package is designed to identify co‐ancestry from RAD data. Haplotypes were run through the fineRAdstructure pipeline using default parameters of 100,000 burn‐in and 100,000 MCMC steps with sampling occurring every 1,000 MCMC steps. A tree was constructed with 10,000 hill‐climbing iterations. Populations were defined as clusters within the fineRAdstructure tree and relatedness plot. The first three axes of variation were used in principal component analysis (PCA) plots. Additionally, factorial correspondence analysis (FCA) plots were generated in GENETIX 4.05.2 (Belkhir, Borsa, Chikhi, Raufaste, & Bonhomme, 2004) from the same SNP dataset.

Pairwise population *F*_ST_ values were estimated among populations inferred from these analyses using arlequin 3.5.1.2 (Excoffier & Lischer, 2010). Also, the contribution of loci under selection on the observed population structure was assessed by identification of *F*_ST_ outlier loci using BAYESCAN 2.1 (Foll & Gaggiotti, 2008) and comparing analyses of structure based on all loci, and excluding outlier loci. Finally, we identified a prevalent *Hin*dIII satellite using nucleotide BLAST (*E *≤* *1 × 10^−10^) against sequenced monomers available in cyprinids *Acrossocheilus paradoxus* (AJ241977.1) and *C. carpio* (M19418.1) and similarly assessed its contribution to population structure.

### 2.6 Population history

DIYABC 2.1.0 (Cornuet et al., 2014) was used to test a number of simple evolutionary scenarios. The dataset was reduced to 661 SNPs by excluding SNPs with missing data for entire populations. One million simulated datasets were generated per scenario at each stage of the scenario testing. We first tested six basic scenarios of a basal split separating one population from all others or a basal polytomy (Figure S7A). We then tested 24 ladder‐like scenarios of successive divergence (Figure S7B). We also tested ten scenarios where the ancestral population split into two major lineages which then diverged into five regional populations (Figure S7C). Finally, we compared the best supported scenarios from the above tests as well as two variants that model the Umgeni population as a product of admixture (Figure S7D). Scenarios were assessed using logistic regression analysis (1% of total datasets) comparing observed versus simulated values of standard summary statistics of genic diversity, population structure, and Nei's distances with all other settings at the default values for SNP datasets.

The average nucleotide diversity across all loci (π = 0.0035) was used to determine the long‐term effective population size (*N*_e_) using the equation from Tajima (1983): π = 4*N_e_*μ. The mutation rate per site per generation (μ) is calculated using a rate of between 1 × 10^−8^ and 1 × 10^−9^ per site per year in SNPs (Brumfield, Beerli, Nickerson, & Edwards, 2003).

## 3 RESULTS

### 3.1 Library preparation and sequencing

Illumina 90‐bp paired‐end sequencing produced over 137 million reads (Table 1) for the 23 individuals. This yielded over 12,027 million total base pairs prior to filtering and trimming, or an average of 523 million base pairs per individual with a range of 372–1,477 million. The average GC content of filtered reads per individual was between 38.5% and 40.8%. The Q20 scores of reads for each individual were within the range of 97.10% and 97.95%, while Q30 was 93.53%–94.81%.

**Table 1:** Summary information from initial analysis of RAD sequencing data

Raw reads	Q20%	Q30%	GC%
137,459,448	97.10–97.95	93.53–94.81	38.5–40.8

### 3.2 Bioinformatics, mapping, and SNP discovery

Initial quality control of the data identified over 52 million high‐quality paired reads without adapter pollution. Mapping reads against the common carp reference genome resulted in an average of 830,241 reads mapped per individual (Table 2). The average coverage of mapped reads was 2.6% of the *C. carpio* genome. In contrast, mapping against *C. carpio* nuclear coding sequences yielded an average number of 55,844 mapped reads per individual with an average coverage of 3.2% of the *C. carpio* CDS reference.

**Table 2:** Mapping of RAD sequencing reads to the *Cyprinus carpio* nuclear (GCF_000951615.1) genome

Mapping	Average reads mapped per individual	Average coverage per individual (%)	Total coverage (%)
*Cyprinus carpio* nuclear genome	830,241	2.6	10
*Cyprinus carpio* entire CDS	55,844	3.2	14

Over 66% of the paired reads were overlapping, indicating inefficient size selection. Additionally, some of the consensus fragments were so short (<90 bp) that sequencing had extended into the adapters and barcode at the 5′ end. Reads contaminated by adapter pollution were filtered with custom scripts using regular expressions based on restriction enzyme recognition sequences. We observed a high degree of incomplete enzyme digestion, with 9% of reads containing more than one of the same restriction site.

Selecting only the first read from each pair gave the current list of 50,740 loci and 17,256 SNPs identified through the stacks pipeline (Table 2). The two methods for identification of potential paralogs, excess haplotypes per individual and Hdplot, produced very different results (see Discussion below). These methods identified 2,435 and 463 loci, respectively (Figure S8). We opted to follow the excess haplotype approach as it listed more putative paralogs. Filtering in populations of mitochondrial loci (121), potentially paralogous loci (2,435), SNPs present in less than 60% of samples (536), SNPs with a MAF of less than 0.04 (1,142), and SNPs recorded as heterozygotes across all samples (125) resulted in a dataset of 826 high‐quality SNPs. This dataset was further reduced to a single SNP per locus based on the number of minor alleles present to accommodate some downstream analysis programs, resulting in the final dataset of 723 SNPs. SNPs found from the initial stacks pipeline and in the final dataset show transition/transversion ratios of 1.58 and 2.04, respectively. The SNP identification process is summarized in Table 3 and distribution of SNPs across individuals in Figure S8.

**Table 3:** Summary of the SNP identification process and filtering steps to the final SNP dataset

Total raw reads	137,459,448
Processed single reads	52,951,112
Loci assembled	50,740
SNPs identified	17,256
Less filters	
Mitochondrial loci	121
Potentially paralogous loci	2,435
SNPs represented across <60% individuals	536
SNPs with MAF < 0.04	1,142
SNPs with *H*_O_ > 0.99	125
SNPs passing all filters	826
One SNP per locus based on number of minor alleles	723

MAF, minor allele frequency; *H*_O_, observed heterozygosity; SNP, single‐nucleotide polymorphism.

### 3.3 Population structure and variation

**Figure 3:**
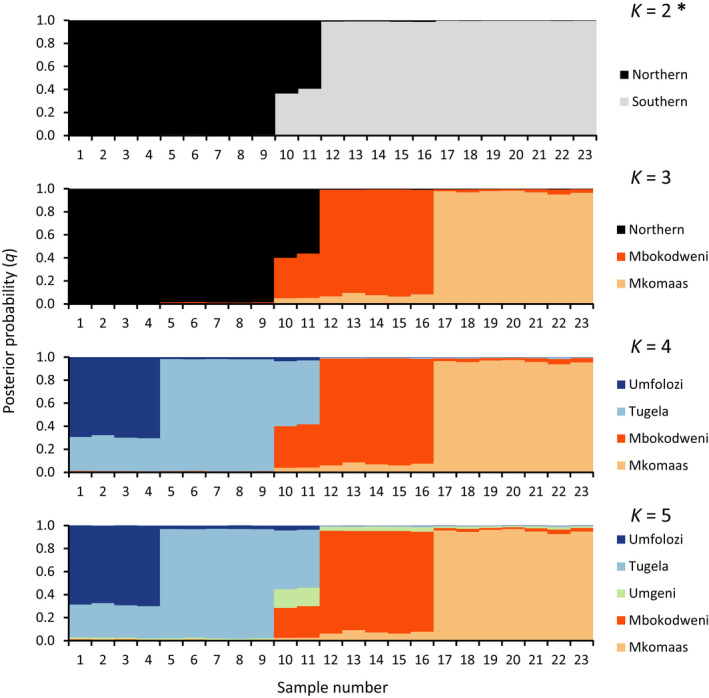
STRUCTURE analysis with *K *=* *2–5 using 723 high‐quality filtered loci. Each individual (indicated as columns along the *X*‐axis) is probabilistically assigned (probability of assignment *q* on the *Y*‐axis) to one of the inferred genetic clusters. Location was specified as prior. *K *=* *2 was recovered as having the most support with the Evanno method (Evanno et al., 2005). CLUMPP was used to produce this representation from 20 replicates

**Figure 4:**
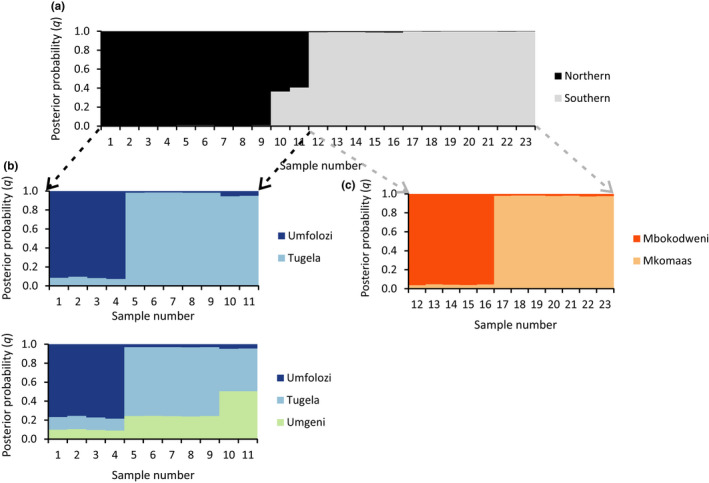
Hierarchical STRUCTURE results for the northern and southern lineages. Each individual (indicated as columns along the *X*‐axis) is probabilistically assigned (probability of assignment *q* on the *Y*‐axis) to one of the inferred genetic clusters. (a) Primary run of STRUCTURE for *K *=* *2 on which the hierarchical STRUCTURE was based. (b) Northern lineage hierarchical STRUCTURE for *K *=* *2 and *K *=* *3 showing the results for minor clusters (4/20 replicates and 6/20 replicates, respectively) only when location is specified as prior. (c) Southern lineage hierarchical STRUCTURE for *K *=* *2 showing the major cluster (14/20 replicates) with location as prior

Analysis of 723 loci with STRUCTURE revealed a well‐supported split at *K *=* *2 between the northern and southern populations (Figure 3; Figures S10 and S11). Individuals from the lower Umgeni (geographically between the Mbokodweni and Tugela drainage systems) appeared as potentially admixed individuals between these northern and southern lineages. Some evidence of further structure at *K *=* *3–5 is present, but this is not as well supported. However, hierarchical STRUCTURE (Figure 4) showed further subdivision into five populations: Umfolozi, Tugela, Umgeni, Mbokodweni, and Mkomaas, from north to south. These results were not well supported (Figures S12–S15). The two samples from the Mzimkhulu system, near the southern distribution limit, were not distinguished from those in the adjacent Mkomaas, and we treat these here as a single population. Similarly, the single sample from Lions River, a tributary of the upper Umgeni, clustered closely with those from the Tugela system, rather than the lower Umgeni, and was considered part of the Tugela population in subsequent analyses (see Discussion below).

**Figure 5:**
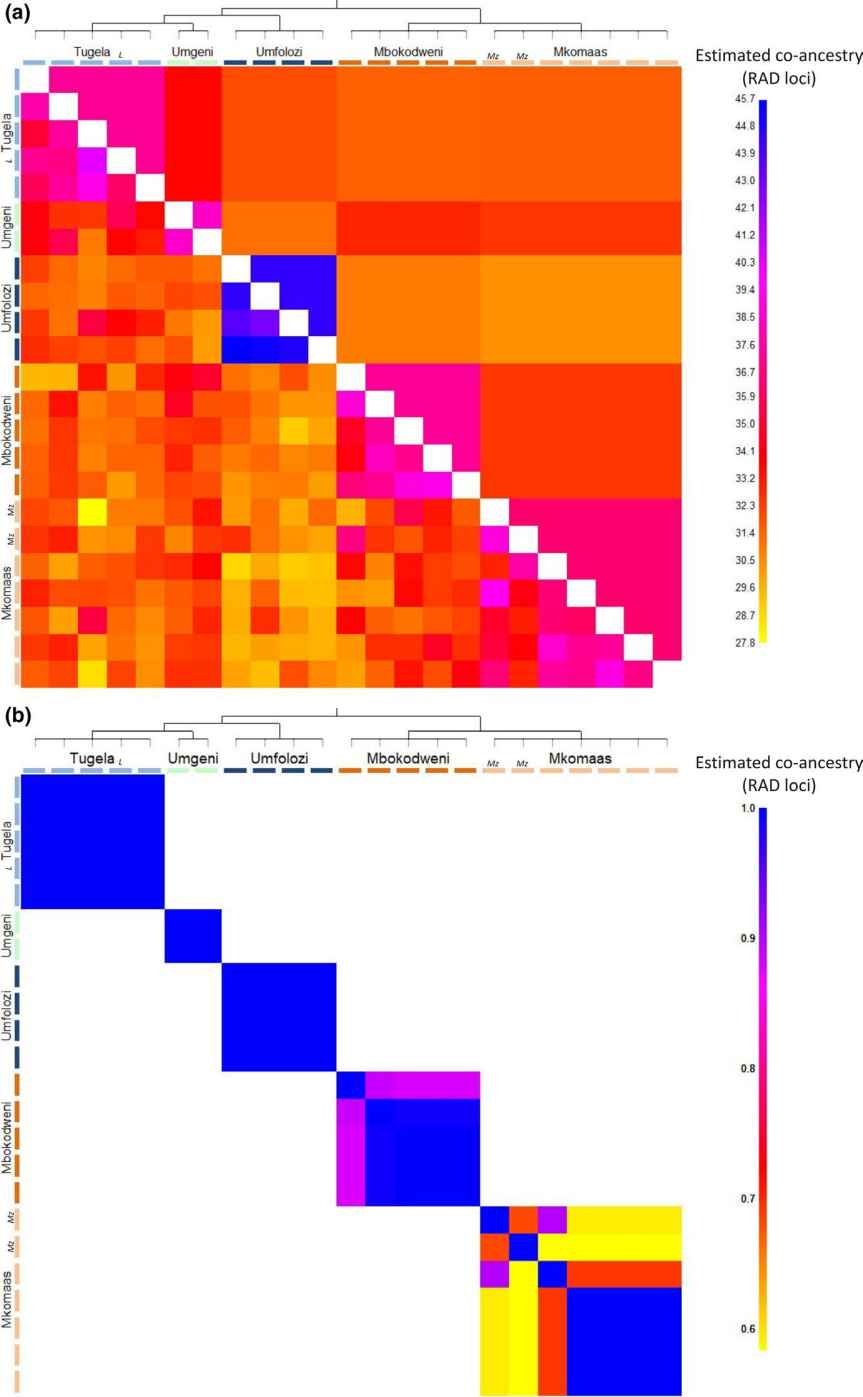
(a) fine RAD structure co‐ancestry matrix, indicating pairwise co‐ancestry between individuals. The lower diagonal indicates raw copy numbers whereas the upper diagonal shows aggregated copy numbers. Individuals clustering into populations are indicated by clustering in the accompanying tree and along the diagonal of the plot. The sample labeled “*L*” is the individual from the Lions River, whereas the samples marked “*Mz*” are the two individuals from the Mzimkhulu. (b) MCMC pairwise comparison heat plot

**Figure 6:**
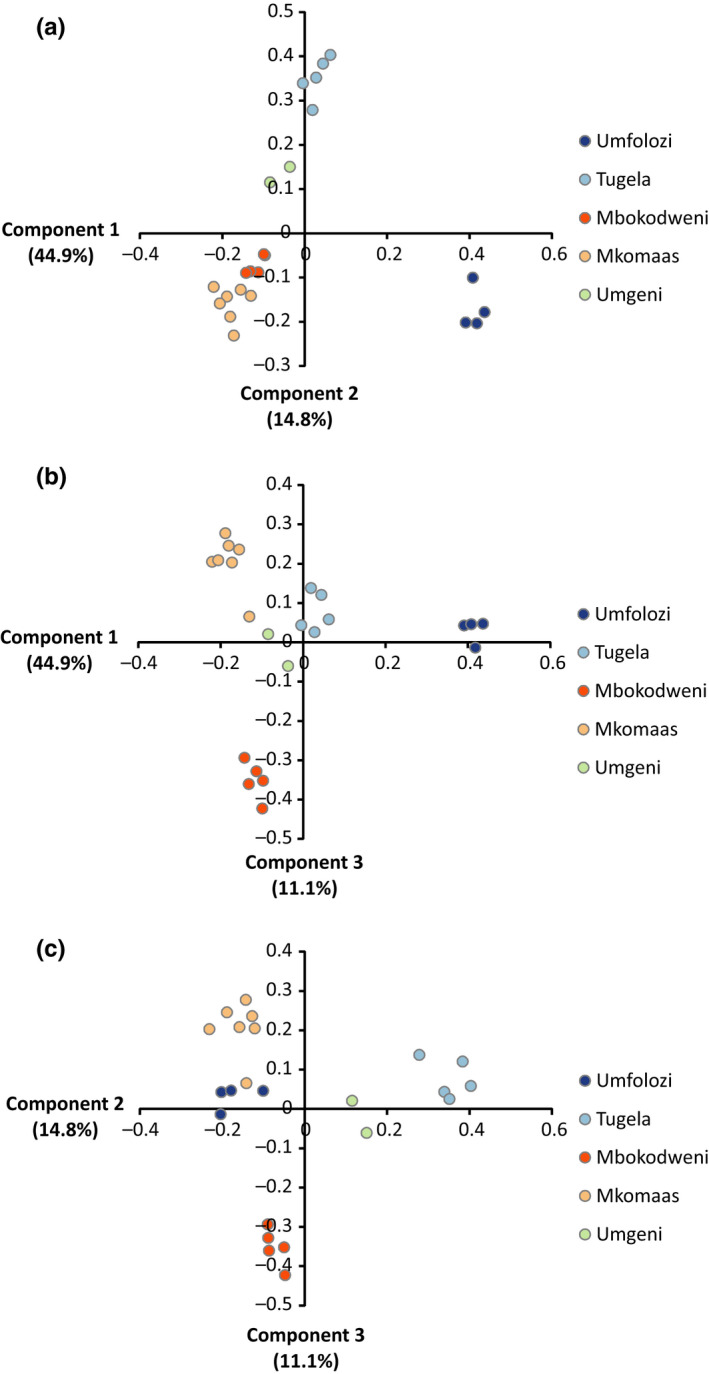
Principal components analysis plots indicating the distribution of individuals into populations according to the first three principal components identified in fine RAD structure, accounting for 70.8% of the eigenvalues. Component 1 splits Umfolozi from the rest. Component 2 splits Tugela (and by extension Umgeni) from all others. Component 3 isolates Mbokodweni from the other populations. (a) The first two components which split samples into all five observed populations. The southern lineage (Mkomaas and Mbokodweni) clusters closely together. (b) Components 1 and 3 split the samples into five populations. Subdivision within the five major populations is most apparent. (c) Components 2 and 3 split the samples into five populations, although one sample of the Mkomaas population (KNU018) clusters closely with the Umfolozi population

**Figure 7:**
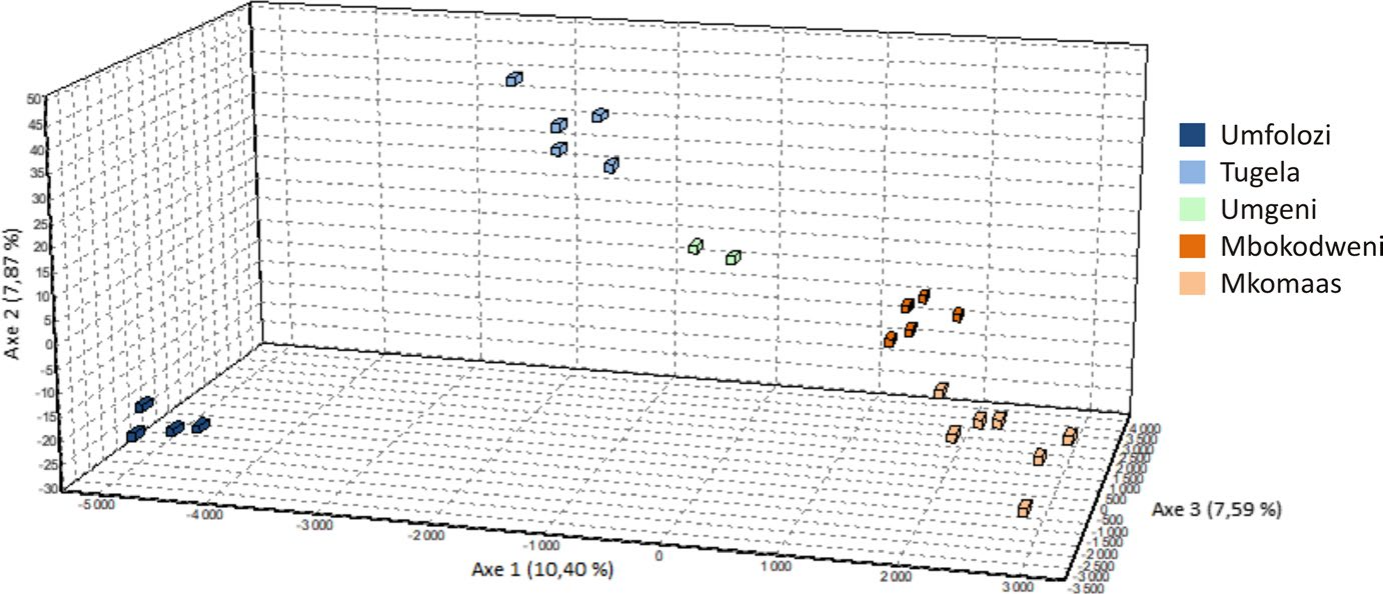
Factorial correspondence analysis plot showing the clustering of individuals into the five populations according to the first three components, accounting for 25.86% of the variance in the data

fineRAdstructure was used to generate a co‐ancestry matrix and tree (Figure 5) showing five populations—the Umfolozi, Tugela, Mkomaas, Mbokodweni, and Umgeni. Further support for these populations was shown in PCA and FCA plots from fineRAdstructure (Figure 6) and genetix (Figure 7). The plots also display variance within populations. The Umgeni was shown to be similar to both neighboring populations (Tugela and Mbokodweni) and was plotted between these two groups.

Positive selection may potentially affect phylogeographic analyses. Consequently, loci in this dataset were filtered for *F*_ST_ outliers using BAYESCAN 2.1 across the five populations using a False Discovery Rate (FDR) of 0.05 and default parameters (20 pilot runs of 5,000 iterations with a burn‐in and final run of 50,000 iterations each). We identified 24 loci as potential *F*_ST_ outliers. However, we did not find any difference in genetic signal when comparing these to other loci, and so all loci were combined for downstream analyses. Removing *Hind*III satellite loci was found to produce similar results, leading to their retention in the final dataset.

**Figure 8:**
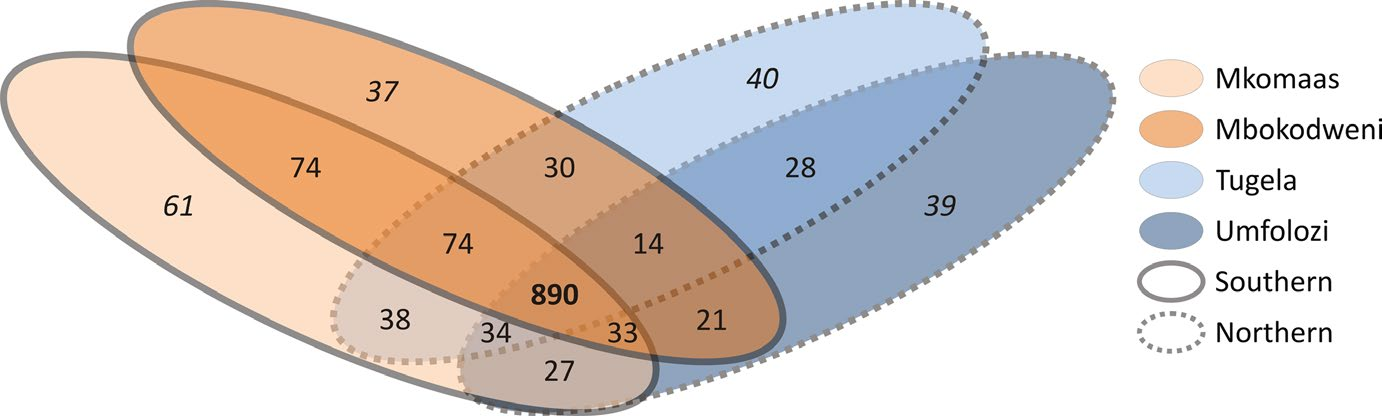
Venn diagram illustrating the association of alleles between four populations (Mkomaas, Mbokodweni, Tugela, and Umfolozi) after samples from the potentially admixed Umgeni population are removed, resulting in 720 diallelic loci. Values in italics indicate private alleles for each of the four populations, whereas the value in bold indicates alleles found in all populations

The number of private alleles varied from 35 in Umfolozi to 57 in the Mkomaas population, except Umgeni which had only six private alleles (Table 4). There was a marked increase of private alleles when the northern (excluding the potentially admixed Umgeni lineage) and southern lineages were specified (107 and 170, respectively). These results show that the northern lineages share 34 alleles which are not found in the southern lineages, and the southern lineages share 77 alleles which are not found in the north. Extending this approach, we removed the potentially admixed Umgeni lineage and categorized each allele as private within a single population, shared between two or more populations only, or shared between all populations (Figure 8).

**Table 4:** Summary data produced by the populations program in stacks for variant positions (top) and all positions (bottom)

	Pop	*N*	Pvt	Sites	% Poly sites	*P*	*H*_O_	*H*_E_	*F*_IS_	π
Variant sites	Umf	3.58	35	533	59.1	0.814	0.370	0.231	−0.179	0.270
	Tug	4.21	38	639	62.4	0.817	0.364	0.232	−0.180	0.266
	Mko	6.17	57	607	71.0	0.806	0.387	0.249	−0.227	0.271
	Mbo	4.22	36	643	65.8	0.800	0.399	0.248	−0.211	0.284
	Umg	2.00	6	492	50.6	0.810	0.374	0.222	−0.118	0.295
	North	7.07	107	719	76.4	0.818	0.357	0.246	−0.186	0.267
	South	9.76	170	720	85.1	0.799	0.392	0.266	−0.234	0.282
	Total	18.5		723	100	0.807	0.373	0.270	−0.218	0.278
All sites	Umf	3.80	35	51,579	0.61	0.9981	0.0038	0.0024	−0.002	0.0028
	Tug	4.65	38	55,557	0.72	0.9979	0.0042	0.0027	−0.002	0.0031
	Mko	6.63	57	55,596	0.78	0.9979	0.0042	0.0027	−0.003	0.0030
	Mbo	4.65	36	54,846	0.77	0.9977	0.0047	0.0029	−0.003	0.0033
	Umg	2.00	6	51,058	0.49	0.9982	0.0036	0.0021	−0.001	0.0028
	North	8.09	107	57,520	0.95	0.9977	0.0045	0.0031	−0.002	0.0033
	South	11.0	170	57,600	1.06	0.9975	0.0049	0.0033	−0.003	0.0035
	Total	21.0		57,840	1.25	0.9976	0.0047	0.0034	−0.003	0.0035

Populations are as follows: Umf, Umfolozi; Tug, Tugela; Mko, Mkomaas; Mbo, Mbokodweni; Umg, Umgeni. Summary data were also calculated for all individuals as a single population (Total) and for the northern (North) and southern (South) lineages identified in this study, excluding the potentially admixed Umgeni lineage.

*N*, average number of individuals genotyped at each locus; Pop, populations; Pvt, number of private alleles; Sites, number of variant sites (top) and total sites (bottom); % Poly Loci, percentage of sites found to be polymorphic; *P*, average frequency of major allele; *H*_O_, average observed heterozygosity; *H*_E_, average expected heterozygosity; *F*_IS_, average Wright's inbreeding coefficient; π, mean nucleotide diversity.

Although the dataset comprises variable loci, only a small proportion of all nucleotide sites (1.25%) were polymorphic. Despite exclusion of singleton SNP loci, the major allele frequency was relatively high across variable sites, suggesting that most loci comprise a common allele and a rare variant. Observed heterozygosity was similar across populations and higher than expected heterozygosity, resulting in negative *F*_IS_ values (see Discussion below). Nucleotide diversity was similar across populations, varying from 0.0028 in Umgeni to 0.0033 in Mbokodweni and 0.0035 overall.

Pairwise *F*_ST_ values between four of the five populations (Table 5) were significant at *p *=* *.01. All pairwise comparisons with Umgeni yielded negative nonsignificant values, probably because this sample comprised two individuals with indications of admixture. The highest pairwise *F*_ST_ values were recorded for the Umfolozi drainage system compared to Mkomaas (*F*_ST_ = 0.057), followed by Mkomaas versus Tugela system (*F*_ST_ = 0.039) and Mbokodweni versus Tugela (*F*_ST_ = 0.039). The lowest positive pairwise *F*_ST_ values were recorded between the two northern populations (Umfolozi and Tugela, *F*_ST_ = 0.002) and the two southern populations (Mkomaas and Mbokodweni, *F*_ST_ = 0.007).

**Table 5:** Pairwise *F*_ST_ values between the five populations identified in this study

	Umfolozi	Tugela	Mkomaas	Mbokodweni
Umfolozi				
Tugela	0.002^a^			
Mkomaas	0.057^a^	0.039^a^		
Mbokodweni	0.032^a^	0.039^a^	0.007^a^	
Umgeni	−0.075	−0.096	−0.043	−0.073

a *p *<* *.01.

### 3.4 Population history

**Figure 9:**
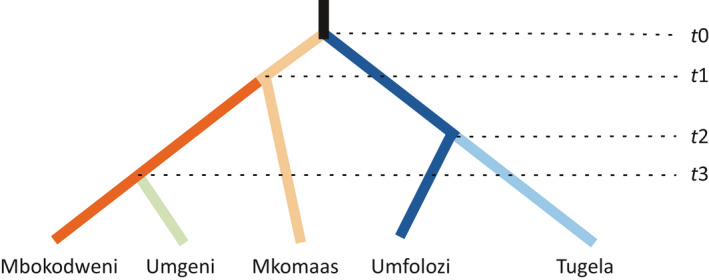
Scenario determined as most likely by DIYABC scenario testing using the logistic regression approach. Divergence times are indicated on the right. Divergence points are not drawn to scale. The node *t*0 was fixed in time as the oldest point, whereas all other nodes were allowed to vary in relation to one another

The best supported scenario from DIYABC analysis involved a split into northern and southern lineages followed by subdivision into three northern and two southern populations (Figure 9). Other scenarios received similar high support, such as a latitudinal series of splits, either from north to south or vice versa, or a split into two northern and two southern populations with admixture in the Umgeni system (Figure S7D). The effective population size is estimated to lie between 87,500 and 875,000 individuals.

## 4 DISCUSSION

### 4.1 Sequencing and mapping

The average GC content of the reads obtained through RAD sequencing, between 38.5% and 40.8%, was similar to that reported for the zebrafish, *Danio rerio*, genome (38.6%) (Zhou, Bizzaro, & Marx, 2004) giving confidence that these data were not particularly biased by our choice of AT‐rich restriction sites (contrary to Campagna, Gronau, Silveira, Siepel, & Lovette, 2015; DaCosta & Sorenson, 2014). The relatively high transition/transversion ratio of 2.04 after filtering for paralogs and mitochondrial loci may indicate a bias toward genic regions, as SNPs occur more frequently as transitions in exons than in introns (Park, Yu, Mun, & Lee, 2010). This value may also reflect effective filters to reduce sequencing error in the final SNP dataset (Pujolar et al., 2013; Rašić, Filipović, Weeks, & Hoffmann, 2014; Zhang et al., 2015). Initial examination of the SNP data, prior to filtering, showed many SNPs in the last few base pairs of reads (Figure S2) that may reflect sequencing errors rather than true variants (Pujolar et al., 2013). Trimming removed most of these errors, as is apparent from the SNP density spectrum (Figure S2), with further errors removed by filtering for singleton allele SNPs and excess heterozygosity.

A general reason for the unexpectedly high genome coverage observed is the paralogous origins of most loci in *L. natalensis* when mapped against lower‐ploidy species such as *C. carpio*. As the hexaploid lineage *L. natalensis* has 150 chromosomes (Oellermann & Skelton, 1990) versus 100 in the tetraploid lineage *C. carpio* (Ráb, Pokorný, & Roth, 1989), a larger amount of the genomic information in the former species originates from shared ancestral sequence duplications. Therefore, the high percentage of mapped reads seen here undoubtedly includes some error from merging of paralogs. Marginally greater coverage was observed in the *C. carpio* nuclear CDS than in the entire nuclear genome, which suggests a bias in the presence of cut sites toward coding sequences, in agreement with the observed transition/transversion ratio. However, this may also reflect a greater likelihood of mapping reads in coding regions, which are more conserved across these distantly related taxa.

The protocol and enzymes chosen for the ddRAD method were estimated to obtain 1.60% of the *D. rerio* genome (Peterson et al., 2012). When the reads obtained for each *L. natalensis* individual were mapped against the *C. carpio* genome, an average of 2.6% coverage per individual was observed. In total, across individuals, reads mapped to about 10% of the *C. carpio* genome. This is despite the distant relation between taxa and the stringent mapping parameters employed to reduce spurious matches. Incomplete enzyme digestion yields loci not accounted for in the estimate of coverage from the *D. rerio* genome. However, less than 9% of sequence reads include additional target restriction sites suggesting that this is only a contributing factor. Although the same fragment size range was targeted as in Peterson et al. (2012), the high proportion of overlap between paired reads indicated imperfect size selection. This may be a common issue with genomic methods involving size selection, as low concentrations of nontarget fragments may be favored by biased amplification and sequencing of short fragments. One consequence of poor enforcement of the size threshold is that a considerably larger portion of the genome was sampled.

### 4.2 Bioinformatics and SNP discovery

A paired‐end ddRAD sequencing approach was used in this study to improve the fragment read depth by avoiding the random shearing step in single‐digest RAD. This method offers the added benefits of requiring less genomic DNA, high repeatability within and among individuals, reduced library construction costs, and allowing highly multiplexed libraries (Peterson et al., 2012). A number of studies have also shown the method to require small sample sizes to produce highly informative population‐level results (Boehm et al., 2015; Macher et al., 2015; Willing, Dreyer, & Van Oosterhout, 2012).

Some predicted drawbacks of this method are the inability of the process to combine stacks of reads as longer contigs, with consequent reduced downstream applications, and potential for bias in estimates of population parameters (Arnold et al., 2013; DaCosta & Sorenson, 2014). In practice, we did not observe these limitations, our mapping showed more extensive coverage than anticipated, and this was reflected in the number of stacks retrieved.

Overall, the method was found to produce relatively low read depths at lower −*m* thresholds (minimum read depth per stack) across the large number of sites identified, probably due to the high frequency of restriction sites for both enzymes, combined with incomplete digestion and size selection. These enzymes were selected to access many loci across the genome. Although efforts were made to restrict fragment size and to achieve complete restriction enzyme digestion, the retention of low levels of nontarget fragments is common to these methods and may have disproportionate influence on the sequence data. An unanticipated by‐product of these technical imperfections, combined with polyploidy of this lineage, is high genome coverage at low read depth. As is typical of many genomic studies, this resulted in a large loss of data through stringent filtering for quality control. In future ddRAD studies, this could be remedied using one rare‐cutting enzyme to reduce the number of fragments sequenced or to increase overall sequencing coverage to improve read depth per site. However, by specifying a minimum depth of five reads per stack, we were able to obtain loci of sufficient coverage (>25×) for population genomic analyses (Paris et al., 2017).

Because coverage is so important for identifying errors from sequencing variation, the substantial variance in loci observed when varying *−m* may indicate that high read depth cutoff should be favored in analyses (Mastretta‐Yanes et al., 2015). However, setting the cutoff value too high would result in allele dropout, leading to further errors (Mastretta‐Yanes et al., 2015). Here, we used the approach of Paris et al. (2017) to determine the optimal stacks parameters by comparing the number of assembled loci, polymorphic loci, and SNPs obtained. This allowed us to generate intuitive graphical representations of how parameters in stacks influenced our dataset (Figure 2; Figures S5–S7; Table S2). From these plots, we chose an optimal set of parameter values for the current dataset.

A large number of paralogous sequences were expected due to the ancestral polyploidy of *L. natalensis*. Beyond the default stacks parameters involved in identifying and filtering potentially paralogous loci, we employed two further approaches: identifying loci with more than two haplotypes in a sample and the Hdplot technique of McKinney et al. (2017). The first approach identified a large proportion of loci as potential paralogs, but this likely also included many loci affected by sequencing error or adapter pollution, which would be removed in any event. In contrast, the Hdplot method was difficult to interpret due to the sparsity of samples (Figure S8), but identified 463 SNPs as potential paralogs. Of these, 42 were not identified by the excess haplotype method or other filters and were retained in the final 723 loci. Although the authors of Hdplot compare an excess haplotype approach to their own method (McKinney et al., 2017), they assessed the haplotypes at the population level, without considering individual diploid genotypes, and therefore did not make full use of these data. Here, we demonstrate that removing loci with excess haplotypes yields more putative paralogous loci and may be more useful for studies with few samples of relatively low coverage. The Hdplot approach is likely still useful as it would identify diverged paralogs, which are fixed for alternate alleles, and should be excluded but which would not yield more than two haplotypes within a single individual (McKinney et al., 2017). Despite our best efforts at isolating paralogous loci, the *F*_IS_ output by stacks reported negative values which are indicative of paralogous loci retained in our dataset (discussed below). This shows that paralogs are resilient to the numerous filtering approaches used here.

Criteria for SNP representation across individuals have a strong effect on the data available for analysis. Recently, there has been a trend advocating the use of datasets with considerable missing data (Buerkle & Gompert, 2013; Chattopadhyay, Garg, & Ramakrishnan, 2014; DaCosta & Sorenson, 2014; Huang & Knowles, 2014; Rubin, Ree, & Moreau, 2012; Wagner et al., 2013), but this has been argued against in other studies (Henning et al., 2014). Initially, we opted for a conservative approach to minimize missing data and thereby reduce uncertainty in population analyses. However, we found that selecting low levels of missing data, at a cost of a smaller dataset, resulted in a loss of power to detect phylogeographic structure (results not shown). As the level of missing data is allowed to increase, so does the signal of phylogeographic structure. This phenomenon has been briefly described in the literature (Campagna et al., 2015; Huang & Knowles, 2014; Takahashi, Nagata, & Sota, 2014; Wagner et al., 2013) and should be viewed as a motivation to include more missing data to reduce potential biases from only examining highly conserved regions. However, our final datasets retained relatively low levels of missing data—19.7% within our 723 SNP dataset and 0.63% within the DIYABC reduced SNP dataset of 661 markers. The difference in missing data observed between these datasets must be due to removing SNPs which contain missing data for entire populations in the latter dataset. This suggests that most missing data we observe are due to mutations within one of the restriction sites leading to locus dropout at the population level (Arnold et al., 2013). These missing data are therefore not due to technical errors or low coverage, but real biological signal from private population mutations.

We found that some signal of finer‐scale structure was being driven by mitochondrial SNPs. STRUCTURE plots generated prior to mitochondrial SNP filtering showed support for higher levels of *K*, depending on the level of missing data allowed (results not shown). This was surprising given that there were only 121 mtDNA markers of 50,740 loci prior to other filters. This highlights the need for effective mitochondrial‐marker filtering of RAD datasets, as these few SNPs influenced signal from all other SNPs. Additionally, we found that the presence of large numbers of singleton SNPs drowned out the signal of genetic differentiation, as observed previously by Rodríguez‐Ezpeleta et al. (2016). This resulted in STRUCTURE plots with no differentiation between populations (data not shown). However, this was resolved by filtering for a minimal MAF set to remove singleton alleles.

Cyprinid genomes include extensive repetitive regions (Henkel et al., 2012) such as the *Hin*dIII satellite (Datta, Dutta, & Mandal, 1988; Tseng, Chiang, & Wang, 2008), which is prevalent in our dataset at a frequency of 4.26% prior to filtering, with 7.47% of the loci in the final dataset potentially affiliated with this satellite. The prevalence of this satellite in our data exceeds that of all satellites across the *C. carpio* genome (2.46%) (Xu et al., 2011). A *Hin*dIII satellite has been shown to exhibit intraspecific concerted evolution in *Cyprinodon variegatus*, whereby it shows low levels of variability within populations and individuals but is distinct between local populations (Elder & Turner, 1994). This is thought to occur either by genetic isolation between populations or by the propagation of new mutational variants across neighboring populations through molecular biological processes such as biased gene conversion (Elder & Turner, 1994). Similar results were recorded in *A. paradoxus* (Tseng et al., 2008) for the satellite sequence to which our *Labeobarbus* satellite matches. In agreement with this, we analyzed a set of *Hin*dIII satellite SNPs independently to our neutral SNPs and found similar results of genetic structure between the populations identified in this study (data not shown). We similarly tested whether the 24 loci potentially under selection identified through BAYESCAN influenced our STRUCTURE results, but found that we obtained the same results whether we excluded these loci or not. As a result, both satellite loci and loci potentially under selection were retained in our final dataset.

### 4.3 Population genetic parameters and structure

Differentiation between groups using STRUCTURE at *K *=* *2 identified a divide between the northern and southern populations. The split between lineages appears to have occurred around Durban. Although this does not coincide with any well‐recognized biogeographic or climatic boundary, it is consistent with a general transition from a speciose tropical fauna, to a highly endemic warm temperate fauna in aquatic organisms (Alexander, Harrison, Fairbanks, & Navarro, 2004; Perera et al., 2011; Seymour, De Klerk, Channing, & Crowe, 2001). Further subdivision into five populations broadly follows the division of KwaZulu‐Natal into the aquatic biogeographic regions of Zululand (Umfolozi), Tugela, Umgeni, and Mzimkhulu (including Mkomaas) (Rivers‐Moore et al., 2007), with the fifth population (Mbokodweni) being more unexpected. Similar divisions within lineages to the biogeographic regions have been observed in freshwater crabs (Gouws, Peer, & Perissinotto, 2015) and in vertebrate fauna overall (Perera et al., 2011).

The division into two broad lineages dominated our primary analysis in STRUCTURE, and subdivision into the five populations is not as well supported. However, further investigation using hierarchical STRUCTURE and fineRAdstructure revealed additional structure. Despite variation among individuals, PCA and independently generated FCA plots also clearly grouped these into five populations, mainly delimited by river systems.

The two individuals from the lower Umgeni drainage system appeared as potentially admixed samples, which may indicate ancestral contact in this system between northern and southern lineages. All *F*_ST_ values for comparisons with Umgeni were negative, suggesting that variance within Umgeni was greater than that between Umgeni and other populations. Unfortunately, only two samples were available from the lower Umgeni, which would affect permutation tests of this result. The Umgeni samples grouped between the neighboring Tugela and Mbokodweni populations in every spatial analysis performed (Figures 6 and 7) which again suggests admixture.

The single sample from the upper Umgeni system (Lions River) consistently grouped closely with those from the neighboring Tugela system (Figures 3, 4, 5, 6, 7). This is likely a translocated individual from the Mooi River, a tributary of the Tugela, via the Mooi‐Mgeni Transfer Scheme (MMTS), an interbasin connection in continuous operation since 2003. We therefore included this upper Umgeni sample in the Tugela population. Similarly, two samples from the Mzimkhulu River near the southernmost point of the distribution were found to consistently cluster with the neighboring Mkomaas River samples as a single lineage, despite grouping as a distinct mtDNA lineage (Bloomer et al. Unpublished data). These samples do not form a distinct group but are responsible for much of the variation within the Mkomaas population, as demonstrated in the fineRAdstructure (Figures 5 and 6) results.

We were not able to include samples from the southernmost limit of distribution, the Mtamvuna River. Similarly, the Mbokodweni River has two neighboring river systems of similar size, the Umlazi and Illovo Rivers which were unsampled, and may contribute to the unexpectedly high diversity found within this population. Further sampling throughout the Umgeni system should be a priority to determine the effect of the ongoing transfer scheme on the upper and lower systems. Other unsampled river systems, including the Umvoti, Amatigulu, and Mhlathuze, are lower priorities for analysis as these medium sized systems are flanked by the larger Tugela and Umfolozi.

The private alleles identified here are a useful resource to distinguish populations. In addition, many alleles shared among the northern populations (excluding Umgeni) were not found in the southern lineages (34 alleles) and vice versa (77 alleles). Further investigation of private alleles after removing the potentially admixed Umgeni lineage revealed a split between the northern and southern lineages and the four populations. The Umfolozi lineage is clearly the most divergent according to the association of alleles between populations as it shares the least out of all populations. *F*_ST_ values also support the deeper split between the northern and southern populations, as pairwise comparisons between groups were consistently higher than those within these groups. Although all efforts were made to remove paralogs from analyses, negative *F*_IS_ values within all populations suggest that some remained in the final dataset. Closely similar paralogs would be combined as allelic variants in stacks, resulting in excess heterozygosity. Where nucleotide substitutions result in consistent differences among paralogs, these combined loci would be excluded by our heterozygosity filter. Difficulties distinguishing interlocus from allelic variation are expected in polyploid species, resulting in excess heterozygosity (Soltis & Soltis, 2000) due to the additional paralogs present.

The identification of five different populations in this study contrasts against the six haplogroups identified using mitochondrial data (Bloomer et al. Unpublished data). This could be due to retention of ancestral polymorphisms at nuclear loci whereas the lower effective population size of the mitochondrial genome would allow population variation fixation at a more rapid rate. Alternatively, this level of fine‐scale differentiation may be beyond the current approach where major historical events could be masking signal from more recent or smaller‐scale events, such as in the case of the primary STRUCTURE analysis where *K *=* *2 was determined to be most likely. However, the most likely explanation for this incongruence is that there is gene flow occurring between these putatively isolated populations such that the finer‐scale nuclear structure within the northern and southern lineages is not substantial, but the mitochondrial locus is rapidly fixed within local populations and reflects a larger degree of difference between these populations. This may suggest a flaw with popular methods such as DNA barcoding, where the sequencing of mitochondrial genes is solely used to delimit populations or even species. Further investigation is necessary to determine whether this is the case here.

### 4.4 Population history

Similar levels of support were received for three models in our scenario testing with DIYABC. The model with the most support matches the results previously observed in our other analyses, where an ancestral population diverged into the northern and southern lineages, which then underwent further subdivision into the current five populations. The ancestral lineage was likely located in Zululand (Umfolozi drainage system), although we cannot rule out the possibility of an Mkomaas ancestral group (Figure S7).

The *N*_e_ produced here is an estimate assuming random mating and constant population size and is associated with the long‐term population size, not the contemporary census size. Parameters of population history were not estimated in DIYABC due to uncertainty of the priors. Because *L. natalensis* is distributed throughout most rivers of KwaZulu‐Natal, it was assumed that the effective population size and hence nucleotide diversity would be correlated with the geographic size of the drainage systems or with the number of associated rivers in a system. This was reflected in the nucleotide diversities for the Umfolozi, Tugela, and Mkomaas systems; however, the Mbokodweni population had nucleotide diversity even greater than the larger systems of Tugela and Mkomaas despite its restricted range of a single known river. Because this is a reflection of the long‐term effective population size, this may indicate that this population historically occupied a more widespread range or could suggest that we have not yet found the full extent of the distribution of this lineage across the coastal rivers in this area. This highlights the need for more comprehensive sampling across this area to define the current range of this population. The current restricted distribution of this population as shown in this study places it as a priority for conservation purposes.

## 5 CONCLUSION

In this study, we used the ddRAD sequencing approach to reduce the genome complexity of a South African endemic hexaploid fish. Our SNP identification protocol was optimized using the new approach of Paris et al. (2017) and extended by comparing two different approaches for paralog identification and removal, filtering of mitochondrial loci which influenced STRUCTURE results, filtering of singleton allele SNPs which masked genetic structure, and retaining satellite loci and loci potentially under selection which both showed similar results to putatively neutral loci. We demonstrated that although a moderate level of missing data was observed, it was due to locus dropout caused by lineage‐specific mutations in one of the two restriction sites. We used our final dataset of 723 SNPs to characterize that two major lineages—northern and southern—diverge into five regional populations—Umfolozi, Tugela, Umgeni, Mbokodweni, and Mkomaas—across the distribution. We found some evidence for north–south admixture within the Umgeni and translocation of a Tugela sample into the Umgeni. Private alleles were identified which support our proposed relationship between the populations. Disparity between previous mitochondrial results and the results presented in this study is likely explained by gene flow between populations. Approximate Bayesian Computation testing suggested a scenario of divergence within the northern and southern lineages into the five current populations. Finally, a number of population genetic parameters are provided in this study including the first estimate of long‐term effective population size and genetic diversity. These indices indicate that the Mbokodweni population may be a key target for conservation efforts. The approaches we used together with the resources established in this study will aid in combating the dearth of genetic data available for *Labeobarbus* and other cyprinids.

## AUTHOR CONTRIBUTIONS

P.B. and C.J.O. designed the research project. P.B., C.J.O., and M.J.C. supervised the research progress. C.S.S. performed research, developed custom scripts, analyzed and interpreted the data, and led the writing of the manuscript. M.J.C. assisted with many of the technical aspects of the project. All authors contributed to the final draft of the manuscript.

## Data Availability

Raw sequence data, processed input files for the final dataset (STRUCTURE, GENEPOP, fineRAdstructure, arlequin, genetix, DIYABC, geste/bayescan, and VCF), and regular expressions for removing adapter pollution are available from the Dryad Digital Repository: https://doi.org/10.5061/dryad.g00b7.
