# Supplementary material for: Exploring the phylogeography of a hexaploid freshwater fish by RAD sequencing
Source: Ecol Evol. 2018 Jan 28;8(4):2326–42. doi: 10.1002/ece3.3821 (PMC5817159; doi:10.1002/ece3.3821)
Supplement: Supplementary file 1 [file ECE3-8-2326-s001.docx]

**Title: Exploring the phylogeography of a hexaploid freshwater fish by RAD sequencing**

Authors: C.S. STOBIE, C.J. OOSTHUIZEN, M.J. CUNNINGHAM AND P. BLOOMER

Molecular Ecology and Evolution Programme, Department of Genetics, University of Pretoria, Private Bag X20, Hatfield, Pretoria, 0028, South Africa

**Supporting Information**

**Table S1** Sampling locations of *L. natalensis* used in this study. Each of the samples listed yielded DNA of a quality satisfactory for RAD sequencing. Sample numbers match those indicated in the STRUCTURE results.

| **Geographic location** | **Drainage system** | **Coordinates** | **Year** | **Samples** | **Sample no.** |
| --- | --- | --- | --- | --- | --- |
| Black Umfolozi River | Umfolozi | -27.93852 S 31.20945 E | 2007 | BLA004; BLU003 | 1, 2 |
| Mkuze River, Farm Verdrukt (444) | Mkuze / Umfolozi | -27.68 S  31.44 E | 2003 | KZF223 | 3 |
| White Umfolozi River, Lenjanedrif, Vryheid | Umfolozi | -27.93503 S 30.95519 E | 2007 | WUR008 | 4 |
| Buffalo River, Tayside | Buffalo / Tugela | -28.05957 S 30.37355 E | 2007 | BUF002; BUF008 | 5, 6 |
| Mooi River, Winterhoek Weir | Tugela | -29.04956 S 30.30382 E | 2006 | MOO007 | 7 |
| Tugela River, Hlalanathi, below DWAF gauging weir | Tugela | -28.65275 S 29.04352 E | 2006 | TUG002 | 8 |
| Lions River | Tugela | -29.42 S  30.01 E | 2003 | KNT212 | 9 |
| Mdloti River, North Coast, Durban | Umgeni | -29.60266 S 31.00874 E | 2006 | MDL003 | 10 |
| Molweni River, Durban Metro | Umgeni | -29.75176 S 30.88918 E | 2006 | MOL004 | 11 |
| Mbokodweni River, Durban Metro | Mbokodweni | -30.00925 S 30.89019 E | 2006 | MBO007; MBO008; MBO009; MBO010; MBO011 | 12, 13, 14, 15, 16 |
| Mkomaas, below Josephine’s Bridge | Mkomaas | -30.01 S  30.15 E | 2003 | KNK315 | 17 |
| Mkomazi River | Mkomaas | -30.13815 S 30.67455 E | 2006 | MKO001; MKO002; MKO003; MKO004 | 18, 19, 20, 21 |
| Mzimkhulu, 2 km above Polela junction | Mzimkhulu / Mkomaas | -29.96 S  29.69 E | 2003 | KNU018; KNB017 | 22, 23 |

**Table S2** Statistics generated as -*n* is varied from -*n* = -*M* – 1 to -*n* = -*M* + 1 using the script count_fixed_snps.py and the populations module of Stacks with a SNP sample representation cut-off of 80% (-*r* = 0.8), as per Paris *et al*. (2017). We followed Paris *et al*. (2017)’s recommendation in selecting the parameter set which yielded the greatest number of polymorphic loci (-*n* = 0).

| **count_fixed_snps.py** |  | | ***m*5*M*1*n*0**  **(-*n* = -*M* – 1)** | ***m*5*M*1*n*1**  **(-*n* = -*M*)** | ***m*5*M*1*n*2**  **(-*n* = -*M* + 1)** |
| --- | --- | --- | --- | --- | --- |
|  | Heterozygous SNPs per sample | BLA004 | 2059 | 2053 | 2033 |
|  |  | BUF008 | 1853 | 1863 | 1857 |
|  |  | MOL004 | 1001 | 1029 | 1069 |
|  |  | MBO009 | 673 | 700 | 725 |
|  |  | KNK315 | 711 | 695 | 688 |
|  |  | MBO008 | 849 | 853 | 870 |
|  |  | MOO007 | 665 | 682 | 699 |
|  |  | TUG002 | 564 | 578 | 598 |
|  |  | MBO007 | 285 | 289 | 299 |
|  |  | KNB017 | 2637 | 2568 | 2544 |
|  |  | BUF002 | 665 | 634 | 605 |
|  |  | KNT212 | 324 | 318 | 311 |
|  |  | KNA017 | 103 | 94 | 93 |
|  |  | WUR008 | 407 | 407 | 411 |
|  |  | MDL003 | 465 | 460 | 467 |
|  |  | KZF223 | 390 | 386 | 388 |
|  |  | MKO001 | 817 | 812 | 802 |
|  |  | BLU003 | 383 | 371 | 366 |
|  |  | MKO003 | 416 | 419 | 417 |
|  |  | BLB004 | 418 | 403 | 387 |
|  |  | KNU018 | 382 | 379 | 382 |
|  |  | MBO010 | 331 | 337 | 330 |
|  |  | MBO011 | 497 | 500 | 494 |
|  |  | MKO002 | 322 | 323 | 320 |
|  |  | MKO004 | 462 | 468 | 462 |
|  | Variable sites across population | | 17679 | 17621 | 17617 |
|  | Heterozygous SNPs in the catalog | | 17679 | 18980 | 21471 |
|  | SNPs across all samples and in catalog | | 17679 | 18634 | 20617 |
|  | Fixed SNPs only in the catalog | | 0 | 1013 | 3000 |
| **Populations**  **(-*r* = 0.8)** | Number of loci | | 2006 | 1982 | 1759 |
|  | Number of polymorphic loci | | ***1198*** | 1185 | 1070 |
|  | Number of SNPs | | 1923 | 1910 | 1645 |


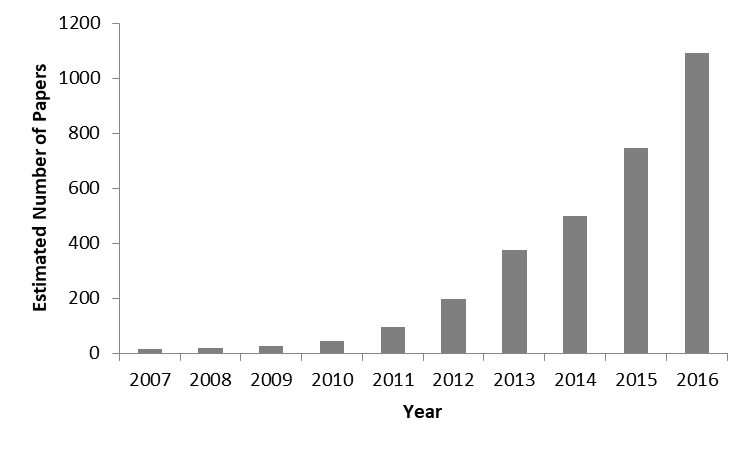


**Fig. S1** Estimated number of research articles using RAD sequencing by year, indicating near-exponential growth. Number of research articles was estimated by the number of Google Scholar hits for “Restriction-site associated DNA”.


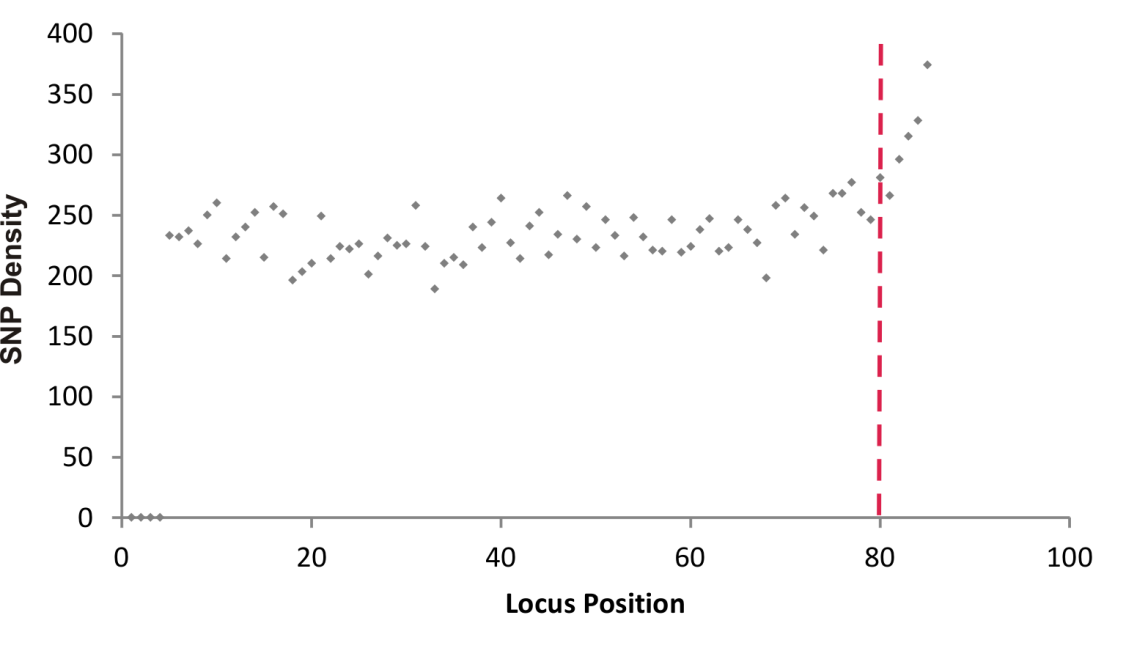


**Fig. S2** SNP density plot for 85-bp Read 1 fragments after initial processing in Stacks using the parameter set -*m* 5 -*M* 2 -*n* 1. The initial uniform distribution suggests low levels of sequencing error, whereas the slight increase in SNPs towards the end of the loci is indicative of sequencing error. Trimming to a length of 80-bp (indicated by the dashed magenta line) removes most of these errors.


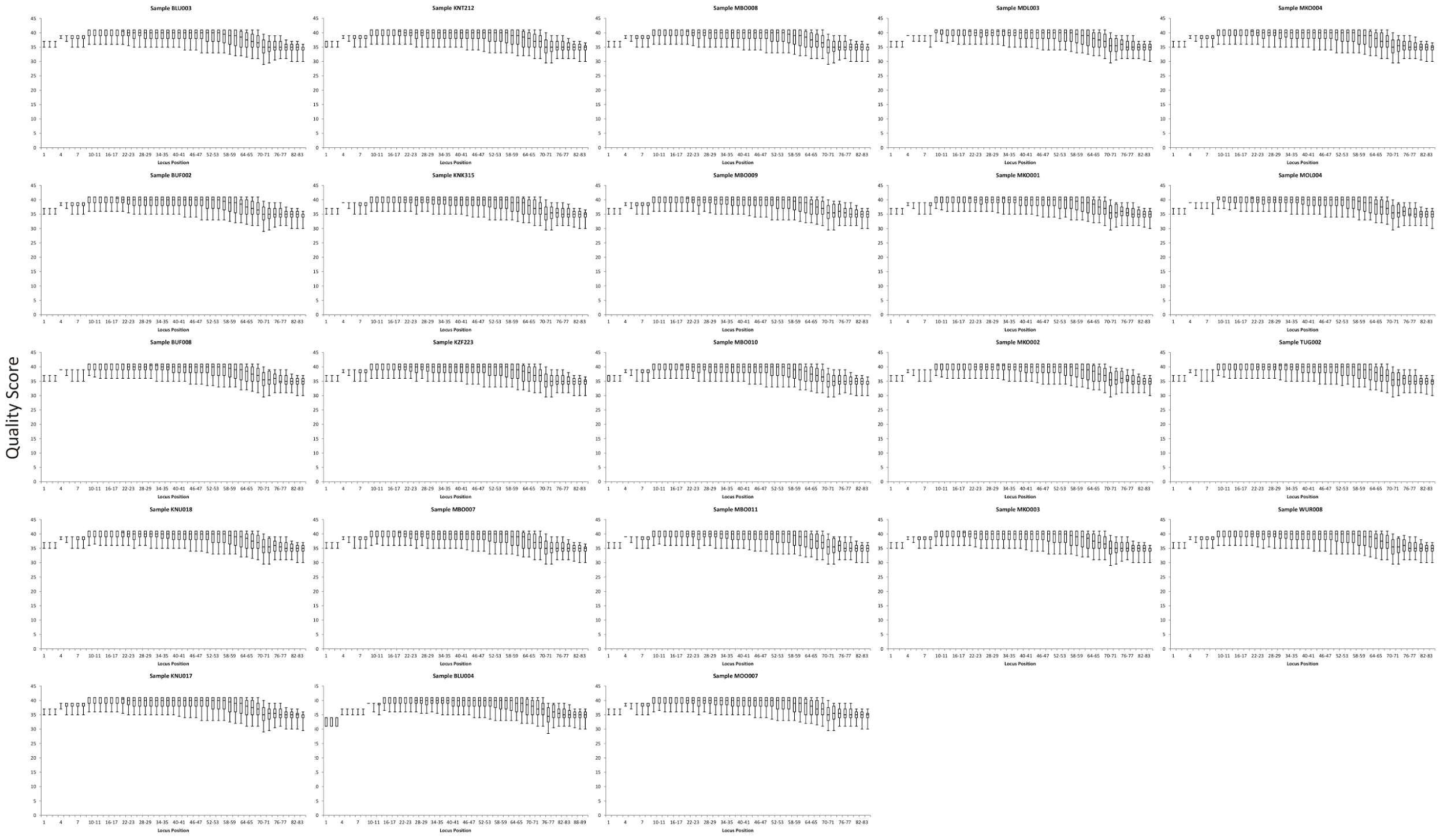


**Fig. S3** Quality scores for each position of the 85-base pair Read 1 sequences of every sample used in this study prior to quality filtering or trimming. Quality scores were obtained using FastQC (Andrews 2010) and are shown using a box-and-whisker plot where the whiskers indicate the 90^th^ and 10^th^ percentiles of the distribution. The quality throughout the positions is high, although this deteriorates towards the end of the reads.


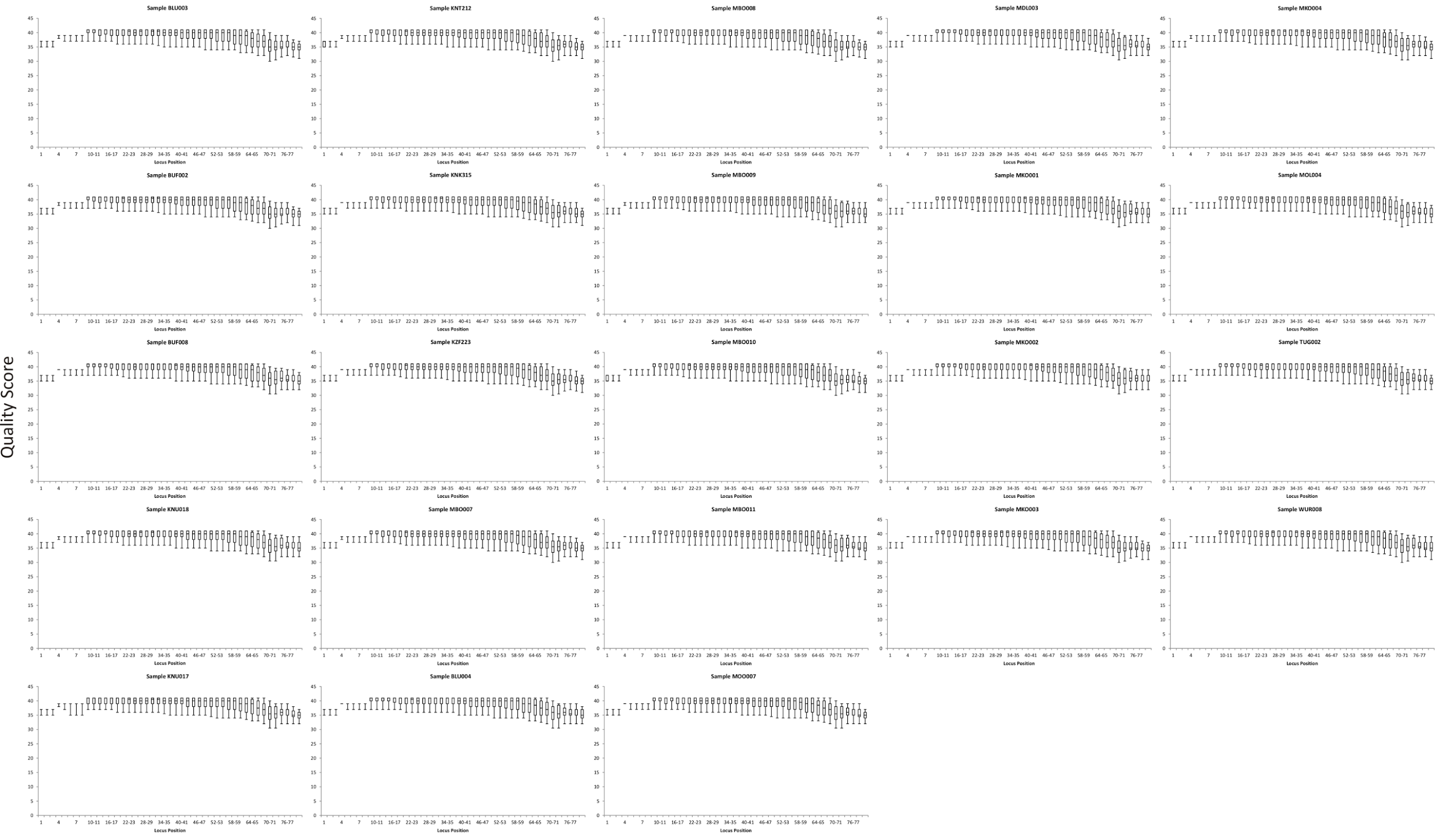


**Fig. S4** Quality scores for each position of the 80-base pair Read 1 sequences of every sample used in this study after trimming and quality filtering in process_radtags. Quality scores were obtained using FastQC (Andrews 2010) and are shown using a box-and-whisker plot where the whiskers indicate the 90^th^ and 10^th^ percentiles of the distribution. The quality is still somewhat diminished towards the end of the reads, but is improved compared to the results prior to trimming.


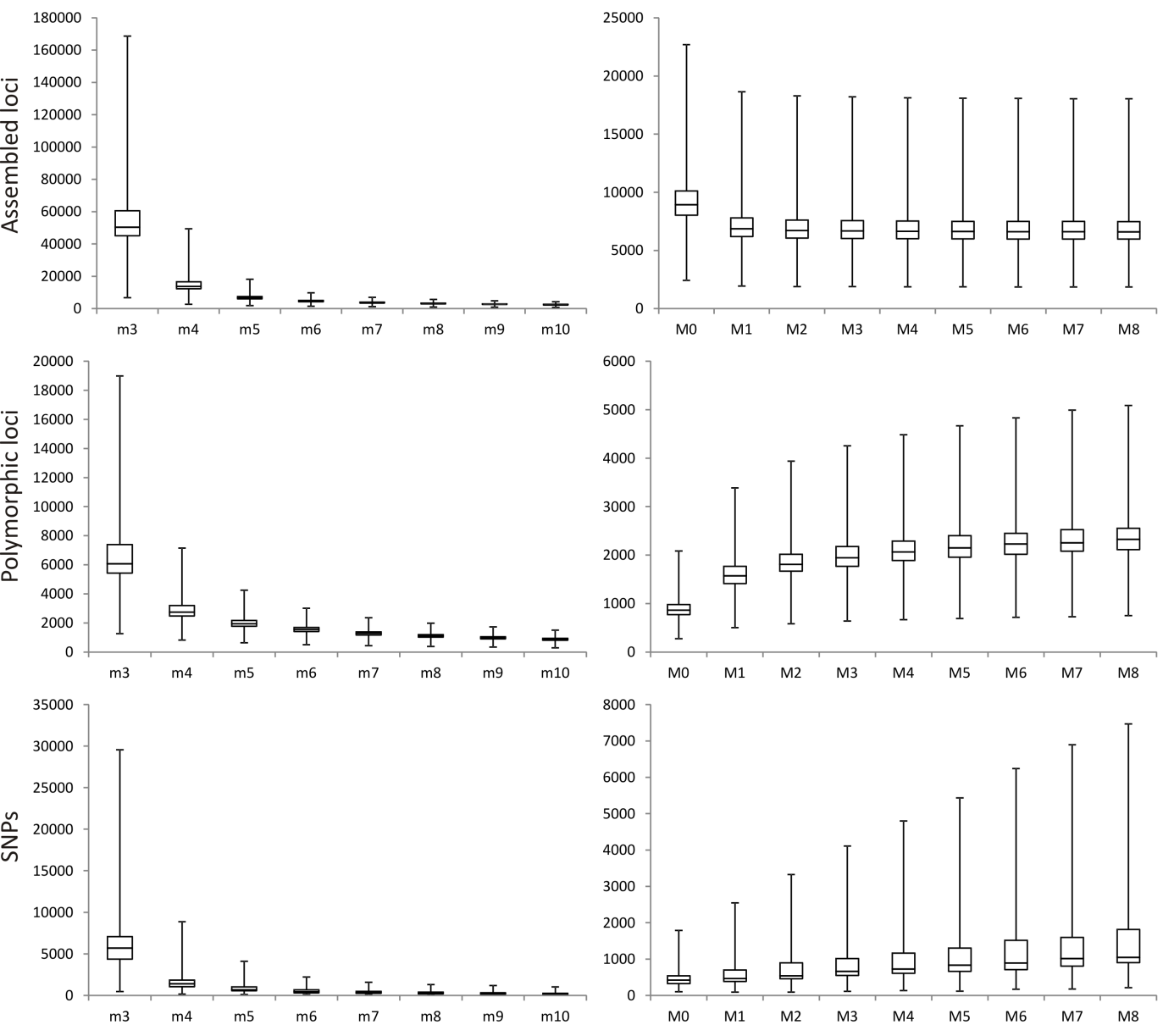


**Fig. S5** Parameter optimisation of the Stacks parameters -*m* and *-M* measured by assessing the total number of assembled loci, polymorphic loci and SNPs identified as the default parameter set (*m*5*M*3*n*2) is changed by the variable on the *X*-axis as per Paris *et al*. (2017). The distribution of results across the 23 samples (plus two replicates) is given by a box-and-whisker distribution where the whiskers indicate maximum and minimum values. The trend for -*M* approaches a plateau at -*M* = 1.


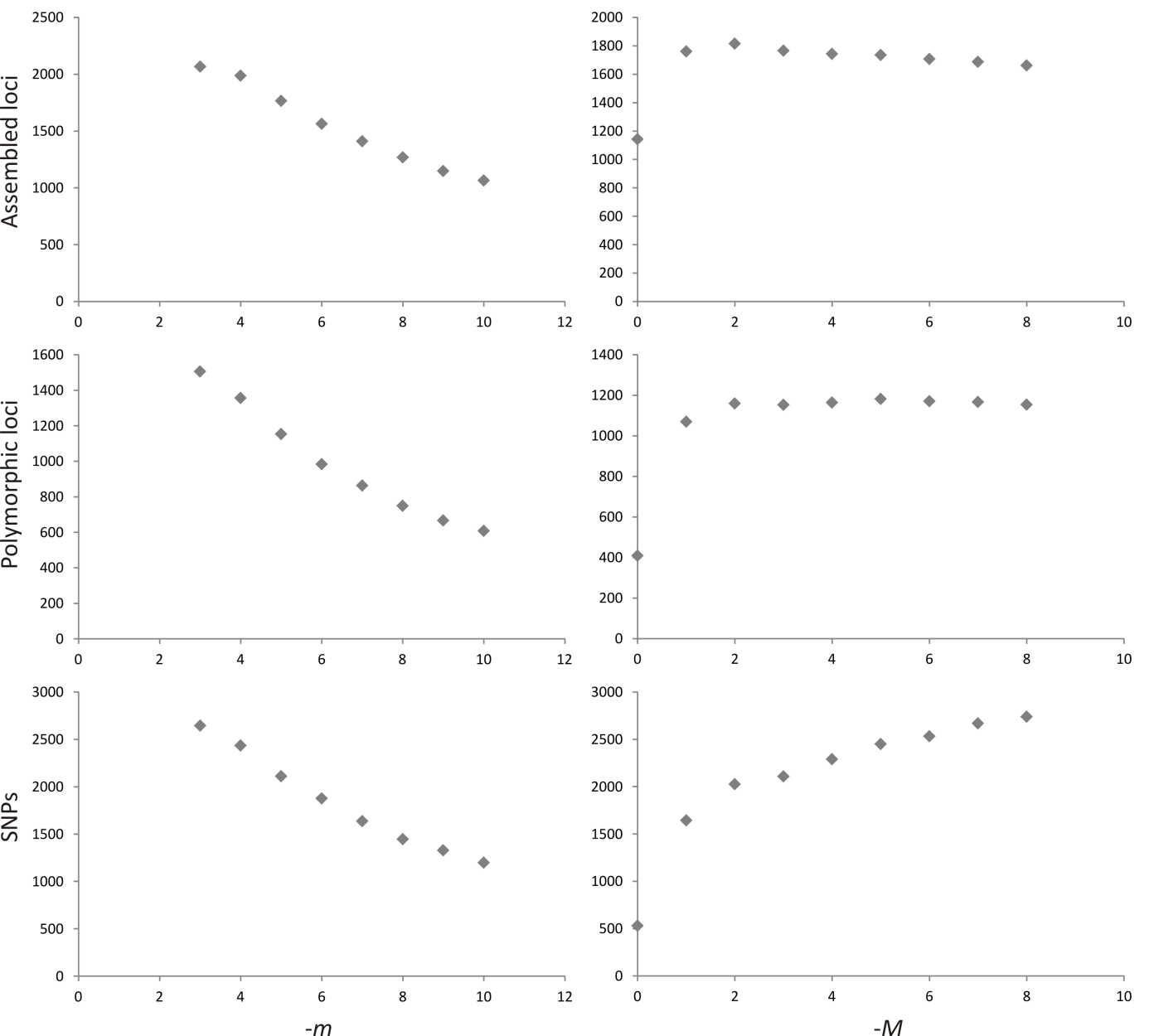


**Fig. S6** Parameter optimisation of the Stacks parameters -*m* and *-M* measured by assessing the total number of assembled loci, polymorphic loci and SNPs identified as the default parameter set (*m*5*M*3*n*2) is changed by the variable on the *X*-axis as per Paris *et al*. (2017). The total values for the entire population (requiring a SNP to be present in at least 80% of samples as per Paris *et al*. (2017)) are indicated. The trend for -*M* again approaches a plateau at -*M* = 1.


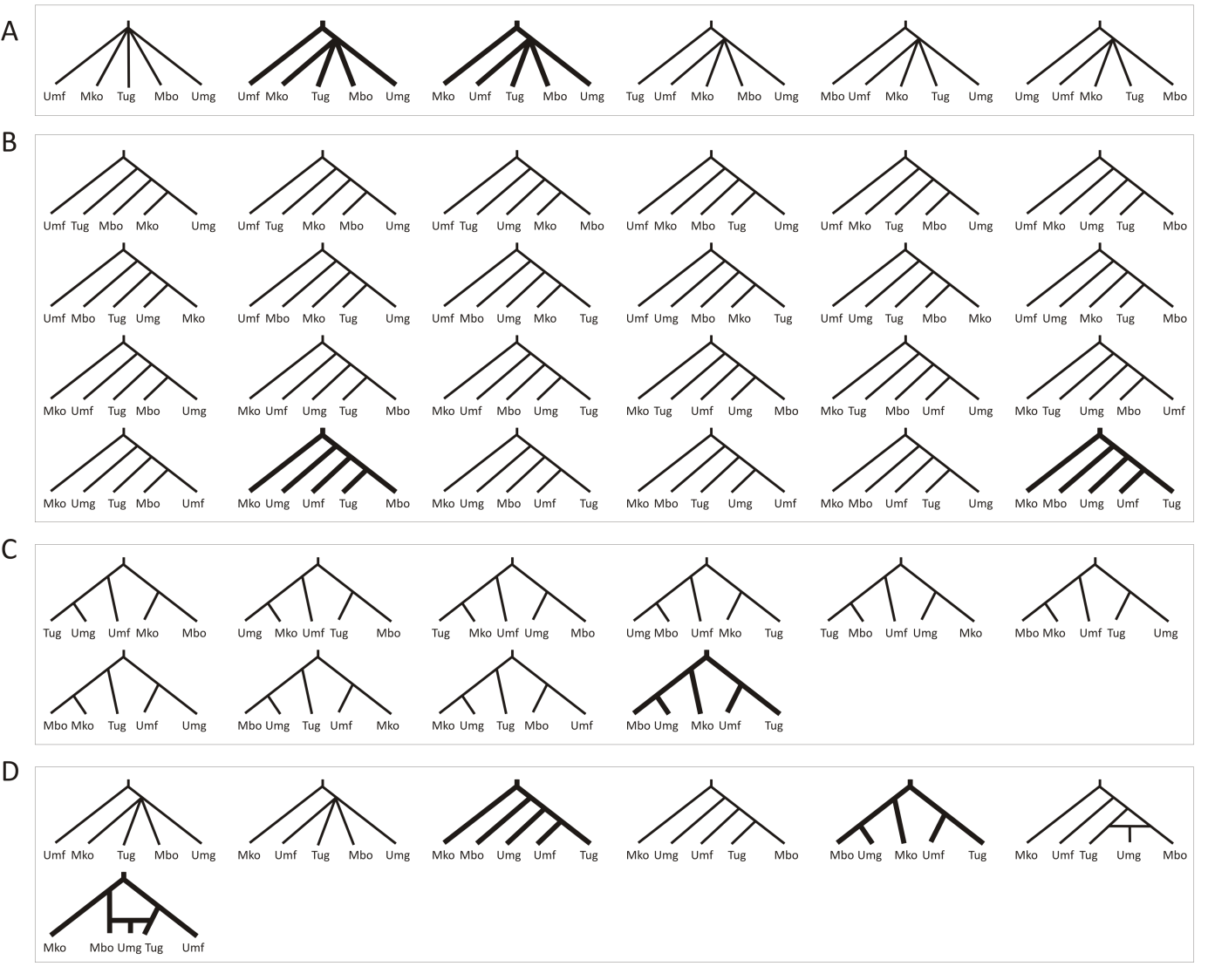


**Fig. S7** All evolutionary pathways tested in DIYABC leading to the current five observed populations. Populations are abbreviated: Mbo = Mbokodweni; Mko = Mkomaas; Umf = Umfolozi; Umg = Umgeni; Tug = Tugela. The scenarios were tested in batches A, B, C and D. Scenarios with the most support using the logistic regression approach for each batch are indicated in bold. A: Basic scenarios of a polytomy were tested. B: Simple evolutionary ladder-like splits resulting in the five populations were tested, keeping the first diverged population as one of the two outgroups identified in A. C: A split into two lineages which later diverged into the current five populations was tested. Note that node ages were allowed to vary, resulting in only the ten scenarios tested. D: All of the most supported scenarios from the previous batches were tested against each other. The fifth scenario was most supported, although scenarios 3 and 7 received very similar levels of support.

**
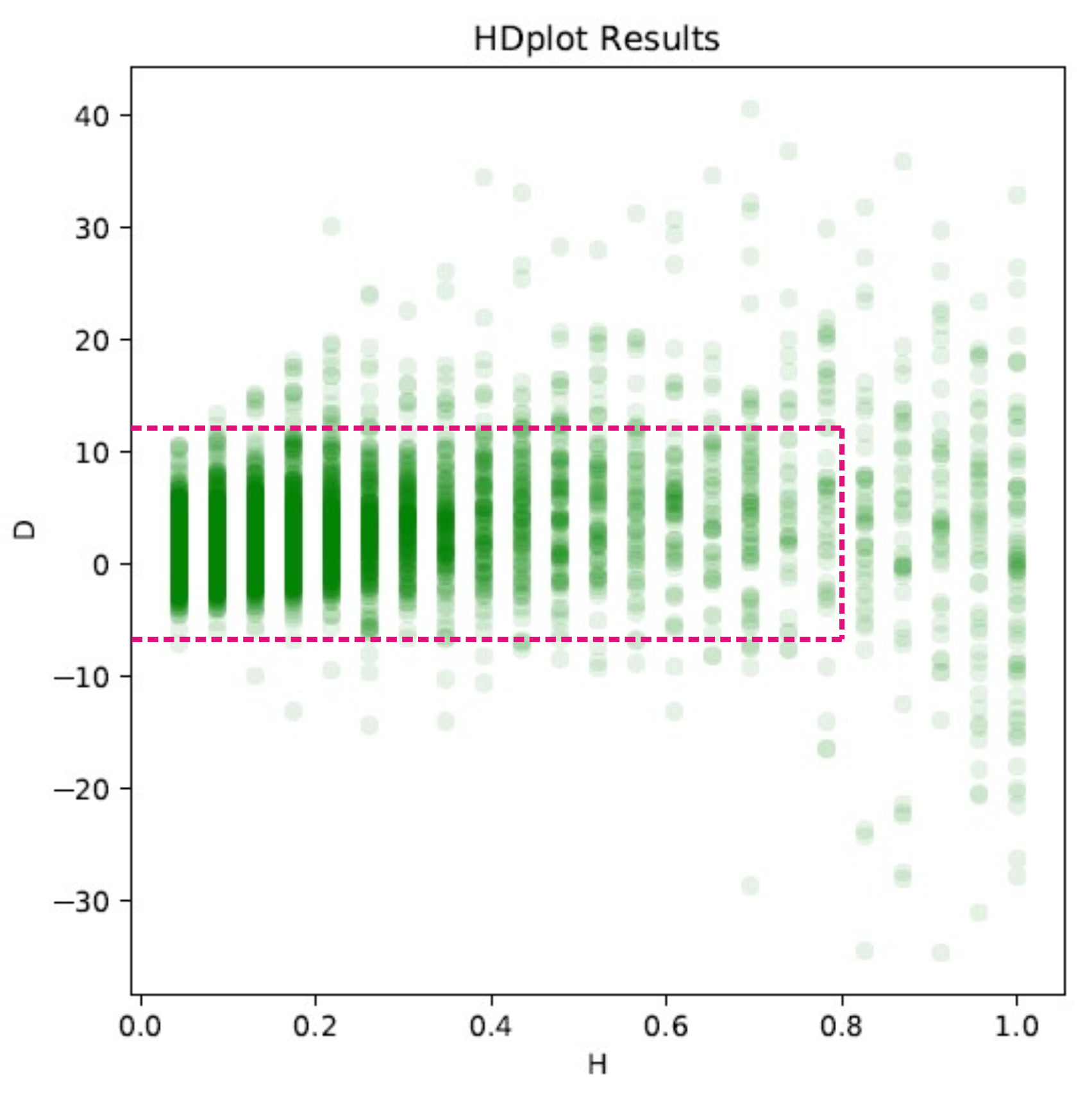
**

**Fig. S8** Results of running the HDplot script (McKinney *et al*. 2017) on the current dataset with no threshold for the number of samples a locus must appear in to be called (-*r* = 0). Due to the sparsity of samples, only a certain number of values are valid for heterozygosity (*H*). The *Y*-axis indicates deviation (*D*) of both alleles of a diallelic locus from 1:1. The primary cloud (indicated by the rectangle) indicates non-paralogous loci, with duplicated loci separating above this cloud and diverged duplicates clustering to the right of the plot. Setting thresholds of *H* < 0.8 and –7 < *D* < 12 yielded 463 of 16,893 SNPs which were identified as potentially paralogous loci.

**
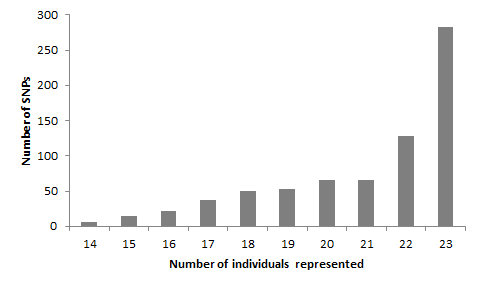
**

**Fig. S9** Distribution of the final set of SNPs across all individuals used in this study due to the requirement for SNPs to be present in at least 60% (=14) of all samples.


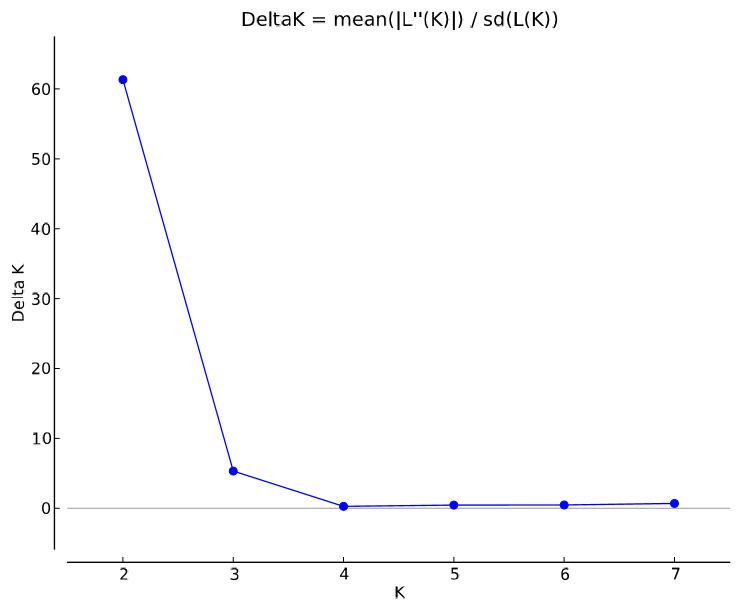


**Fig. S10** STRUCTUREHarvester results showing Δ*K* across *K* = 1-8 for the primary dataset prior to hierarchical STRUCTURE (Fig. 3).


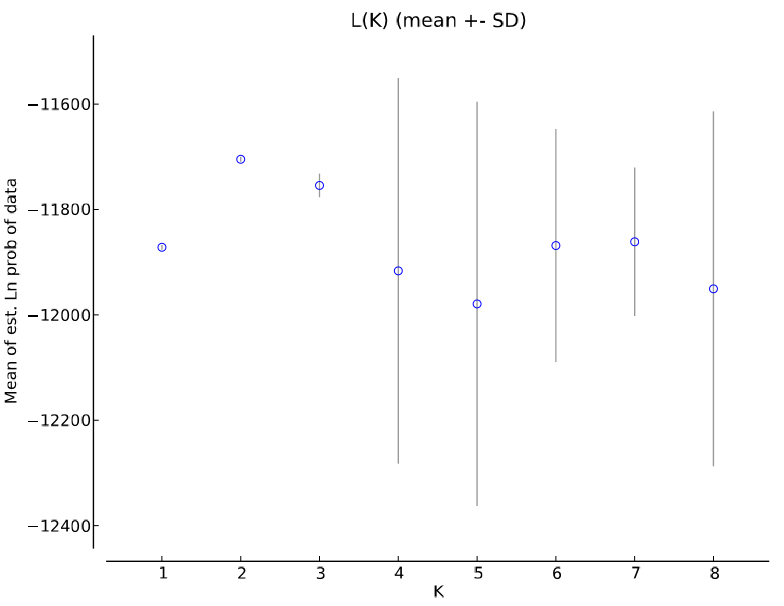


**Fig. S11** STRUCTUREHarvester results showing likelihood *L* (*K*) across *K* = 1-8 for the primary dataset prior to hierarchical STRUCTURE (Fig. 3). Standard deviation is indicated by the bars extending from each point.


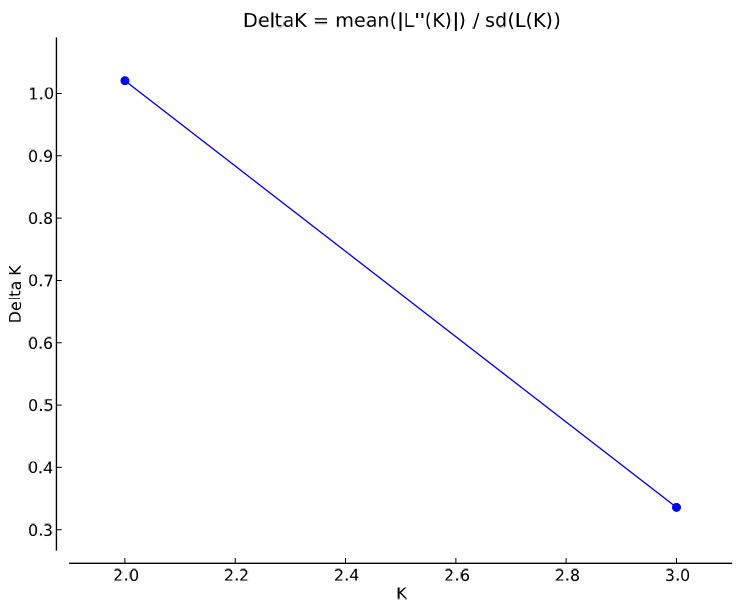


**Fig. S12** STRUCTUREHarvester results showing Δ*K* for the northern lineage only across *K* = 1-4 in the hierarchical STRUCTURE run (Fig. 4).


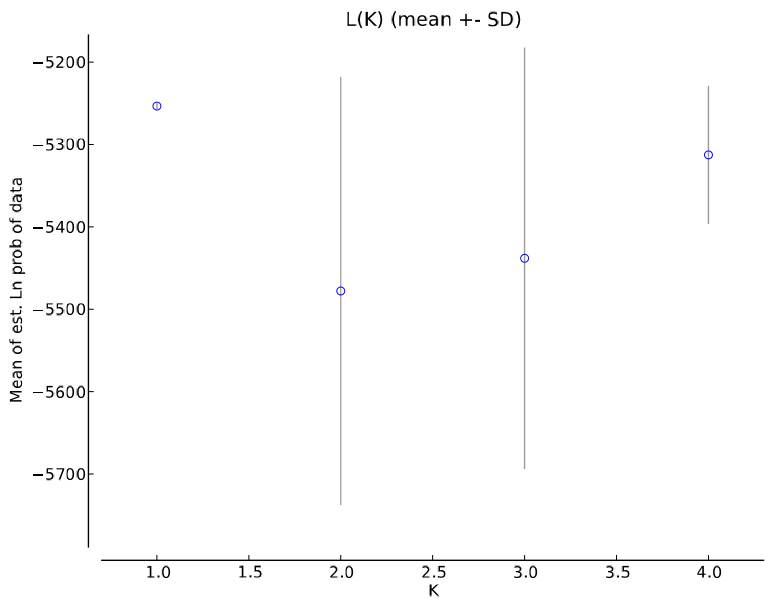


**Fig. S13** STRUCTUREHarvester results showing likelihood *L* (*K*) for the northern lineage only across *K* = 1-4 for the hierarchical STRUCTURE run (Fig. 4). Standard deviation is indicated by the bars extending from each point.


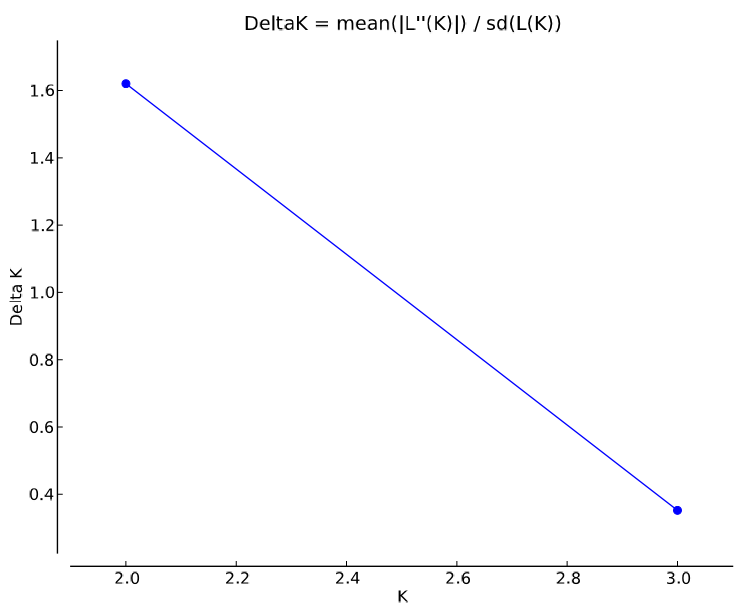


**Fig. S14** STRUCTUREHarvester results showing Δ*K* for the southern lineage only across *K* = 1-4 in the hierarchical STRUCTURE run (Fig. 4).


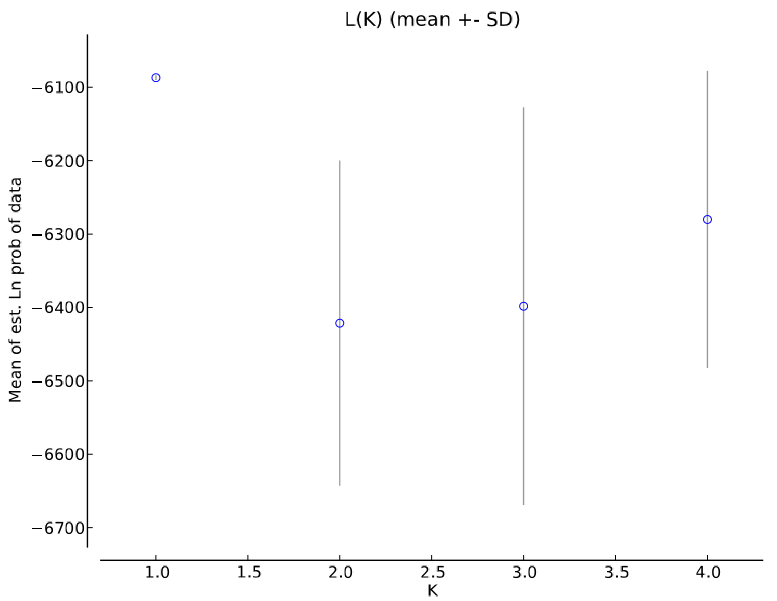


**Fig. S15** STRUCTUREHarvester results showing likelihood *L* (*K*) for the southern lineage only across *K* = 1-4 for the hierarchical STRUCTURE run (Fig. 4). Standard deviation is indicated by the bars extending from each point.

**References**

Andrews S (2010) FastQC: a quality control tool for high throughput sequence data. Available online at: <http://www.bioinformatics.babraham.ac.uk/projects/fastqc>.

McKinney GJ, Waples RK, Seeb LW, Seeb JE (2017) Paralogs are revealed by proportion of heterozygotes and deviations in read ratios in genotyping‐by‐sequencing data from natural populations. *Molecular Ecology Resources* **17**, 656-669.

Paris JR, Stevens JR, Catchen JM (2017) Lost in parameter space: a road map for stacks. *Methods in Ecology and Evolution* **8**, 1360-1373.
